# SIGLEC15 modulates the immunosuppressive microenvironment and suppresses malignant phenotypes in triple-negative breast cancer

**DOI:** 10.1016/j.gendis.2025.101799

**Published:** 2025-08-09

**Authors:** Zhaofu Tan, Hongbin Xin, Jian Chen, Ming Lei, Gang Tu, Lingfeng Tang

**Affiliations:** a Department of Breast and Thyroid Surgery, Chongqing Key Laboratory of Molecular Oncology and Epigenetics, The First Affiliated Hospital of Chongqing Medical University, Chongqing 400010, China; b Department of Dermatology and Venereology, The First Affiliated Hospital of Chongqing Medical University, Chongqing 400010, China

**Keywords:** Breast cancer, Epithelial‒mesenchymal transition, SIGLEC15, T cell suppression, Tumor microenvironment, ZEB1

## Abstract

Previous studies have demonstrated a significant association between sialic acid binding Ig-like lectin 15 (*SIGLEC15)* and both the progression of malignant tumors and immune infiltration. This study comprehensively analyzed and elaborated the function and related mechanism of *SIGLEC15* in breast cancer. We analyzed *SIGLEC15* expression levels and predicted its functions using mRNA sequencing in a population-based dataset. Single-cell RNA sequencing was utilized to investigate the biological roles of *SIGLEC15* within the tumor microenvironment (TME). Finally, we conducted both *in vivo* and *in vitro* experiments to validate the findings derived from the RNA sequencing analyses. Elevated *SIGLEC15* expression was associated with favorable outcomes in breast cancer patients. Tumor cells exhibiting high *SIGLEC15* expression demonstrated reduced epithelial–mesenchymal transition (EMT) tendencies compared to those with lower expression levels, potentially through the regulation of *ZEB1* expression. However, anti-tumor immunity was significantly suppressed in the TME containing these tumor cells. Analysis of protein expression in patient samples revealed a negative correlation between *SIGLEC15* expression and CD4, CD8 T-cell infiltration. In mouse models, tumor cells overexpressing *SIGLEC15* exhibited diminished invasive and migratory capabilities. Furthermore, both *in vitro* and *in vivo* experiments confirmed that Nutlin-3a has a more pronounced inhibitory effect on breast cancer cells with elevated *SIGLEC15* expression. The expression level of SIGLEC15 can serve as a biomarker to assess the malignancy of breast cancer and the degree of immune infiltration. Monitoring SIGLEC15 expression levels can facilitate more informed and personalized clinical decision-making for the treatment of breast cancer patients.

## Introduction

Breast cancer (BRCA) ranks first in incidence among female carcinomas and has become increasingly prevalent and severe in recent years.^1^ Although well-established treatments for BRCA are widely applied in clinical settings, they often come with significant side effects.^2^, ^3^, ^4^ For patients diagnosed with BRCA before tumor metastasis, therapies such as surgery, endocrine therapy, and targeted therapy can significantly improve survival rates.^1^ However, for patients with advanced tumor metastasis, immunotherapy has emerged as an effective approach to prolong survival. However, the efficacy of immunotherapy in triple-negative breast cancer (TNBC) remains heterogeneous.^3^^,^^5^

In the tumor microenvironment (TME), immune cells play a crucial role not only in the response to immunotherapy but also in chemotherapy, both in adjuvant and neoadjuvant settings, across all BRCA subtypes.^6^^,^^7^ Unfortunately, this promising new therapy faces numerous challenges, including various forms of drug resistance.^8^ Therefore, it is imperative to identify novel therapeutic targets that can be widely applied in clinical practice to benefit more patients.

The epithelial–mesenchymal transition (EMT) is a reversible process in which epithelial cells lose their characteristic properties and acquire mesenchymal traits, accompanied by altered expression levels of cell adhesion molecules and cytoskeleton components.^9^, ^10^, ^11^ EMT is a dynamic and continuous spectrum of transitions, during which cells lose apical polarity, gain anterior‒posterior polarity, reduce cell adhesion, shift from an epithelial to a mesenchymal phenotype, and acquire mesenchymal properties.^10^^,^^11^ This process has been shown to promote cell migration and invasion,^12^ providing a critical perspective for understanding tumor metastasis.

SIGLEC15 (sialic acid-binding immunoglobulin-type lectin 15) is an emerging immunosuppressive transmembrane protein that is highly expressed in various solid tumors, including pancreatic cancer, thyroid cancer, bladder cancer, and glioma. It promotes tumor progression by shaping an immunosuppressive TME^13^, ^14^, ^15^, ^16^. A previous study has demonstrated that SIGLEC15, when expressed on tumor-associated macrophages (TAMs), exhibits an M2-like phenotype. It interacts with tumor cells in an α-2,3-linked sialic acid-dependent manner, activates the SYK phosphorylation pathway, promotes immunosuppressive cytokine secretion, accelerates tumor growth, and impairs CD8^+^ T cell function.^17^ In colon adenocarcinoma (COAD), elevated SIGLEC15 expression in the stromal compartment is significantly correlated with poor clinical outcomes and remains an independent prognostic factor irrespective of PD-L1 expression.^18^ Notably, SIGLEC15 and PD-L1 exhibit a complementary expression pattern. In hepatocellular carcinoma (HCC) and bladder cancer (BLCA), elevated SIGLEC15 expression is correlated with a non-inflammatory TME and confers resistance to immunotherapy.^15^^,^^19^^,^^20^ Mechanistically, the immunosuppressive function of SIGLEC15 is mediated through N172 site glycosylation. Genetic ablation of this modification site markedly suppresses tumor growth *in vivo*^14^. These findings identify SIGLEC15 as a promising therapeutic target for cancer immunotherapy. In breast cancer, SIGLEC15 is overexpressed in both tumor cells and tumor-infiltrating immune cells, where its expression is correlated with advanced disease stage, lymph node metastasis, and unfavorable clinical outcomes.^21^ Mechanistically, SIGLEC15 promotes bone metastasis by accelerating osteoclast differentiation and impairing bone homeostasis.^22^ Preclinical evidence demonstrates that anti-SIGLEC15 antibody therapy significantly reduces the metastatic burden and extends survival. Notably, SIGLEC15 and PD-L1 display mutually exclusive expression patterns, positioning SIGLEC15 as a particularly promising immunotherapeutic target for PD-L1-negative patients.^23^ Contrary to its established role as a prognostic marker in luminal breast cancer, we discovered that SIGLEC15 exhibits dual functionality in TNBC, simultaneously regulating tumor biological behavior and immune modulation within the TME.

This study aimed to explore the functions of *SIGLEC15* in BRCA. Using RNA sequencing datasets from public databases, we inferred the biological roles of *SIGLEC15* in both the TME and tumor cells. Through bioinformatics analyses, we further validated these functions through *in vivo* and *in vitro* experiments. It is anticipated that our study will contribute to the development of more personalized treatment strategies for clinical breast cancer management.

## Materials and methods

### Data collection

We collected data from multiple online databases, including the Genomics Expression Omnibus Database (GEO), The Cancer Genome Atlas Program (TCGA), and Genotype-Tissue Expression (GTEx). TCGA is a comprehensive web-based resource featuring diverse datasets, such as gene expression profiles, methylation patterns, and copy number variations, from over 11,000 tumors across 33 cancer types, along with their corresponding clinical data. For this study, we accessed RNA-sequencing data (level 3) for the breast invasive carcinoma cohort from the TCGA and the GTEx databases. This dataset included tissue samples from 1109 cases, clinical features from 1097 patients, and 113 matched adjacent normal tissue samples (data retrieved up to January 1, 2020). The TCGA data were accessed via https://portal.gdc.cancer.gov/, and the GTEx data were retrieved from https://www.genome.gov/Funded-Programs-Projects/Genotype-Tissue-Expression-Project. The RNA-sequencing data, initially presented as Fragments Per Kilobase of exon model per Million mapped fragments (FPKM) values, were converted to Transcripts Per Kilobase Million (TPM) values for standardized analysis.

We also utilized several GEO datasets (GSE176078,^24^ GSE190772,^25^ GSE162228,^26^ and GSE42568^27^) accessible at https://www.ncbi.nlm.nih.gov/geo/. Single-cell RNA sequencing (scRNA-seq) datasets were analyzed using the Chromium System by 10X Genomics. Specifically, GSE176078 contains scRNA-seq data from primary BRCA tumor samples; GSE190772 contains scRNA-seq data from BRCA metastases to bone.

### Tumor heterogeneity analysis

The relationships between homologous recombination deficiency (HRD), loss of heterozygosity (LOH), and *SIGLEC15* expression in pan-cancer cohorts were evaluated using the online tool SangerBox,^28^ accessible at http://sangerbox.com.^28^

### Tumor gene mutation landscape and expression analysis

We performed Chi-square tests to evaluate differences in gene mutation frequencies between the high and low *SIGLEC15* expression groups within the TCGA-BRCA cohort. The top 15 significantly mutated genes were identified and analyzed for differential expression patterns.

### Immune cell infiltration analysis

The Estimate^29^ (v1.0.13) and CIBERSORT^30^ (v0.99.9) R-packages were used to assess differences in stromal and immune cell infiltration between the high and low *SIGLEC15* expression groups in TCGA-BRCA.^29^^,^^30^

### Functional enrichment analysis

In our study, we utilized the FindMarkers function from Seurat^31^ v4 to identify differentially expressed genes (DEGs) across various groups within single-cell datasets. The selection criteria for DEGs included an adjusted *P*-value of less than 0.01 and a log2 fold change greater than 1.

For pathway analysis, we focused on genes with differential expression across *SIGLEC15* expression groups, particularly those influenced by *SIGLEC15* expression in the TCGA-BRCA dataset. Genes with an adjusted *P*-value of less than 0.05 and a log2 fold change exceeding 1 were selected for further analysis. Pathway enrichment was performed using HALLMARK gene sets, with visualizations generated via the SangerBox online platform. Only pathways with a false discovery rate (FDR) below 0.1 were considered statistically significant.

In the context of gene set variation analysis (GSVA),^32^ we applied GSVA scoring to pathways using GO gene sets and TPM data from bulk transcriptome sequencing datasets, including TCGA-BRCA, GSE162228, and GSE42568. To identify significantly enriched pathways, we employed stringent criteria, including an adjusted *P*-value threshold of less than 0.01 and a minimum log2 fold change of 1.4.

For pathway enrichment analysis of single-cell data, we used the irGSEA R-package (v1.0.13). Pathway enrichment was assessed using HALLMARK gene sets^33^ across individual cells, employing tailored methods such as AUCell, UCell, singscore, and ssGSEA to mitigate background noise. Differences in pathway enrichment scores across clusters were analyzed using the Wilcoxon test, with an adjusted *P*-value threshold of less than 0.05 for significance. To integrate the results across various analyses, we employed the RobustRankAggreg (RRA) method using the RobustRankAggreg R-package (v1.1.0). This approach allowed us to isolate consistently enriched pathways across multiple gene set enrichment methodologies. The entire process was conducted via the irGSEA R-package to ensure a comprehensive and robust pathway enrichment analysis.

### Single-cell sequencing analysis

The single-cell RNA sequencing data utilized in this study are available in the GEO database under the accession numbers GSE176078 and GSE190772. The datasets were processed and analyzed using Seurat v4. For GSE176078, the quality control thresholds included a minimum of 150 genes per cell (minGene), a maximum of 5500 genes per cell (maxGene), a maximum of 15,000 unique molecular identifiers (maxUMI), less than 20% mitochondrial gene content (pctMT), and less than 1% hemoglobin gene content (pctHB). Similar quality control criteria were applied to GSE190772.

To identify and remove potential doublets, we employed the DoubletFinder package (v2.0.3). All the samples from the two datasets was integrated using the Harmony package (v2.0.3), ensuring consistency across different samples and enabling robust downstream analysis.

### Cell differentiation analysis

We employed the monocle3 package (v1.3.1) and CytoTRACE^34^ (v0.3.3) to estimate the differentiation trajectories and the differentiation potential of malignant cells derived from BRCA bone metastatic tumors.

### Cell–cell interaction analysis

We used the R-package CellChat^35^ to investigate the interactions of the TME cells between different groups.

### Hub gene filtered analysis

We used the high-dimensional Weighted Gene Co-expression Network Analysis (hdWGCNA)^36^ to filter the hub genes between different single-cell *SIGLEC15* expression groups. The soft power is seven (Fig. S1C).

### Transcription factor inference

We used the R-package decoupleR^37^ to infer the transcription factors in the single-cell RNA sequence dataset GSE176078.

### Drug resistance prediction

We used the R-package oncopredict^38^ (version 0.2) to estimate the normalized FPKM data of TCGA-BRCA to predict the drug IC_50_ in patients.

### Cell culture

The MCF10A breast tissue cell lines, MCF7, T47D, BT474, SKBR3, BT549, MB468, and MB231 and the HEK293T human embryonic renal cell line were purchased from the American Type Culture Collection (ATCC, Beijing, China). MCF10A and BT549 cells were cultured in Park Memorial Institute-1660 (RPMI-1640, Gibco) supplemented with 10% fetal bovine serum (FBS, Gibco, 10099141) and 1% penicillin and streptomycin (P/S). The Lenti-X 293T cells and the breast cancer cell lines were maintained in Dulbecco’s modified Eagle’s medium (DMEM, Gibco) supplemented with 10% FBS (Gibco, 10099141), 1% P/S, 4 mM l-glutamine, 1 mM sodium pyruvate, 0.1 mM nonessential amino acids (MEM), and 0.5 mg/mL G418. All the cell lines were cultured in 5% CO_2_ at 37 °C in a humidified incubator.

### Transfection and infection

To construct the lentiviral cDNA overexpression vector, human SIGLEC15 cDNA (NCBI, NM_213602) and human ZEB1 cDNA (NCBI, NM_001323638) were amplified via PCR and cloned into the LV-ECMV-PURO vector, which was modified from pCDH-CMV-MCS-EF1a-CopGFP-T2A-Puro (System Biosciences, USA). For lentivirus production, Lenti-X 293T cells were seeded in 10 cm dishes 24 h prior to transfection. A mixture of 2 μg pMD2.G (Addgene, 12259, USA) envelope vector, 4 μg pspAX2 (Addgene, 12260, USA) packaging vector, and 5 μg lentiviral vector with 1 mL optiMEM (Gibco, USA) and 20 μL Lipofectamine 3000 (Thermo Fisher, USA) transfection reagent were then added to the cells and incubated for 20 min.

The medium was refreshed 6 h after transfection, and the supernatant of the 293T cells containing lentivirus was collected 48 h after transfection. For stable overexpression cell line construction, viral supernatant supplemented with 8 μg/mL polybrene (Beyotime, China) was used to transduce BT549 and MB231 cells, followed by selection with 1 μg/mL puromycin for 7 days (Beyotime, China). Then the method for transfecting SIGLEC15-knockdown MB231 is similar to that used for overexpression, with the target sequences for the SIGLEC15 shRNAs being 5′-GCAAGTGGAACCTGTACAT-3′ (sh1), 5′-GCAGATCAACTTCCACATCA-3′ (sh2), 5′-GCTGGTGGAGATCATCGTGAA-3′ (sh3), and 5′-GCTCCTACACCAACATCCTT-3′ (sh4). And the control shRNA sequence that did not match any known human cDNA was 5′-AAGGTGTCAGAAACTGACGAT-3′. The protein expression of SIGLEC15 was analyzed by Western blotting after transfection.

### Clinical pathological specimens

Human tissue microarrays of formalin-fixed paraffin-embedded (FFPE) breast tumors and normal patient tissues were obtained from The First Affiliated Hospital of Chongqing Medical University. This study received approval from the Ethics Committee of The First Affiliated Hospital of Chongqing Medical University (No. K2023-514).

### Immunohistochemistry (IHC)

Human breast tissue was fixed in 4% paraformaldehyde overnight, embedded in paraffin, and sectioned into 5 μm. Human FFPE tissues were deparaffinized in xylene and rehydrated through graded ethanol solutions (100%, 95%, 70%, and 50% EtOH). Antigen retrieval was performed at 95 °C–100 °C in 10 mM sodium citrate buffer with 0.05% Tween-20, pH 6.0, for 15 min. Endogenous peroxidases were quenched using 3% H_2_O_2_ for 10 min, and were blocked in 5% BSA, 5% normal goat serum, and 0.1% Tween-20 in TBS for 1 h. Primary antibodies were diluted in blocking buffer and incubated overnight at 4 °C in a humidified chamber. After being washed with 0.1% TBS-T, the slides were incubated with SignalStain Boost IHC Detection Reagent (Cell Signaling Technology, 8114) for 30 min at room temperature. After washing three times with 0.1% TBS-T, the signal was developed using the ImmPACT DAB Substrate Kit (Vector Laboratories, SK4105). The slides were counterstained with hematoxylin and then dehydrated through reverse-graded ethanol solutions (50%, 70%, 95%, and 100% EtOH), cleared in xylene, and mounted with DPX mounting media (Millipore Sigma, 317616). The following primary antibodies were used with the indicated dilutions: rabbit anti-SIGLEC15 (Abcam ab198684, 1:100), rabbit anti-CD4 (Servicebio GB11064-100, 1:200), rabbit anti-CD8 (Servicebio GB115692-100, 1:200), rabbit anti-PD-1 (Servicebio GB12338-100, 1:200), rabbit anti-FOXP3 (Proteintech No. 22228-1-AP, 1:200), rabbit anti-CD69 (Servicebio GB115670-100, 1:200), and rabbit anti-CXCR1 (Servicebio GB11625-100, 1:200).

### Wound-healing assay

A straight line was scraped with 10-mL pipette tips from cells grown in 6-well plates. After the plates were washed twice with PBS, the cells were incubated in an FBS-free medium. Microscope photographs of the wound healing made in the BT549 and MB231 cells were taken at 0 h and 24 h. The wound width at 0 h was set to be 100% in each group. A wound width analysis was performed with Photoshop and GraphPad 5.0 software.

### Cell migration and invasion

A total of 75,000 breast cancer cells in 150 μL DMEM medium were added to each insert with an 8 μm pore size (Falcon, 353097), and 800 μL of complete growth medium supplemented with 10% FBS was added to each well on a 24-well cell culture plate. After 24 h, the cells were fixed with 100 % methanol for 20 min at room temperature. Then, the transwells were stained with 0.5% crystal violet in a 20% methanol solution for 20 min. The remaining crystal violet and non-invading cells on the transwells were removed with a cotton swab. Subsequently, the transwells were washed three times with distilled water for 5 min. To monitor cell invasion ability, 150 μL of 1 mg/mL Cultrex Reduced Growth Factor Basement Membrane Extract (R&D Systems, 3433-005-R1) was prepared in advance to add to the transwells, and were incubated at 37 °C for 30 min. Images were obtained, and the cells were counted using ImageJ with a customized macro. Briefly, a binary mask was applied to the images, and the analyze particle function was used to count invading cells with a minimum size of 300 pixels and minimum circularity of 0.05 to avoid detecting the scale bar.

### Western blot (WB) assay and antibodies

For the WB assays, the cells were harvested and lysed with RIPA lysis buffer containing protease inhibitor cocktail for 30 min. Then, the samples were centrifuged for 15 min (12,000× *g*, 4 °C), and the supernatant was collected. After boiling, the proteins in the supernatant were separated by 10% SDS-PAGE and transferred to a polyvinylidene fluoride (PVDF) membrane. The PVDF membrane was blocked with 5% fat-free milk for 2 h at room temperature and then incubated with primary antibodies (anti-SIGLEC15 (1:1000; Proteintech), anti-ZEB1 (1:1000; Proteintech), anti-ZEB2 (1:1000; Proteintech), anti-N-cadherin (1:1000; Proteintech), anti-E-cadherin (1:1000; Proteintech), anti-Vimentin (1:1000; Covance), anti-Slug (1:1000; Abcam), anti-Snail1 (1:1000; Proteintech), anti-Snail2 (1:1000; Proteintech), anti-Cyclin D1 (1:1000; Proteintech), anti-Cyclin B1 (1:1000; Abcam), anti-Cyclin E1 (1:1000; Abcam), anti-E2F1 (1:1000; Abcam), anti-CDK4 (1:1000; Proteintech) and anti-β-tubulin (1:10000; Sigma)) overnight at 4 °C. The following day, after being washed three times with TBST buffer, the membrane was incubated with secondary antibodies for 2 h at room temperature. The signals were detected by enhanced chemiluminescence (ECL) reagent (Clinx Science, Shanghai, China) and visualized by WB detection instruments (Clinx Science, Shanghai, China).

### Cell proliferation assay

The cell viability assay was performed using Cell Counting Kit-8 (MCE, China) according to the manufacturer’s instructions. The cells were seeded in 96-well plates at a density of 5000 cells/well and cultured overnight before drug additions. The cells were incubated with DMEM containing 10% CCK8 solution and incubated at 37 °C for 2 h, after which the absorbance was measured at 450 nm using an enzyme-labeling instrument (Thermo Fisher Scientific, USA).

### Colony formation assay

BT549 and MB231 cells were plated into 6-well plates at 1500 cells/well. The media were replaced every 3 days, and the cells were allowed to grow for 10–14 days. Paraformaldehyde was used to fix the cells, 0.2% crystal violet solution was used for staining, and photographs were taken.

### Xenograft studies

All animal experiments were approved by the Ethics Committee of Chongqing Medical University (IACUC-CQMU-2023-0267). Four-week-old female athymic BALB/c-nu mice, purchased from Vital River Laboratories (Beijing), were maintained until 5 weeks of age in the specific pathogen free (SPF) animal facility of the Laboratory Animal Resource Center of Chongqing Medical University.

A total of five million SIGLEC15-overexpressing or vector-transfected MB231 cells were resuspended in a 1:1 solution of PBS and BD Matrigel® at high concentration (BD Bioscience, USA), and were injected orthotopically into the fourth pair of mammary fat pads of the nude mice. For the phenotype experiment, tumor-bearing mice were randomized (using the RAND function in Microsoft Excel) into the Nutlin-3a and control groups (saline solution) as indicated (*n* = 5/group). The statistician was blinded to the treatment group allocation. After 2 weeks, Nutlin-3a or saline solution was administered intraperitoneally in a corn oil solution at a dosage of 60 mg/kg per week. For the *in vivo* assay of SIGLEC15-knockdown MB231 cells, carboplatin (MedChemExpress) was administered via intraperitoneal injection at 60 mg/kg according to the manufacturer’s instructions, with the control groups receiving saline solution as indicated (*n* = 5/group).

Based on the measurement of the Vernier caliper, the tumor volume was assessed via the modified ellipsoidal formula: tumor volume = ½ length × width.^2^ When the tumor grew to about 100 mm^3^, The mice were euthanized by cervical dislocation after an overdose of anesthesia at the experimental endpoint.

### Statistical analysis

All the statistical analyses were performed in R (version 4.3.2). Nonparametric tests (Wilcoxon signed-rank test for different *SIGLEC15* expression groups) were used to compare the cell proportions between different groups. *P* < 0.05 was considered to indicate statistical significance, and all the statistical tests were two-sided.

## Results

### Higher *SIGLEC15* is related to better prognosis in BRCA patients

**Figure 1:**
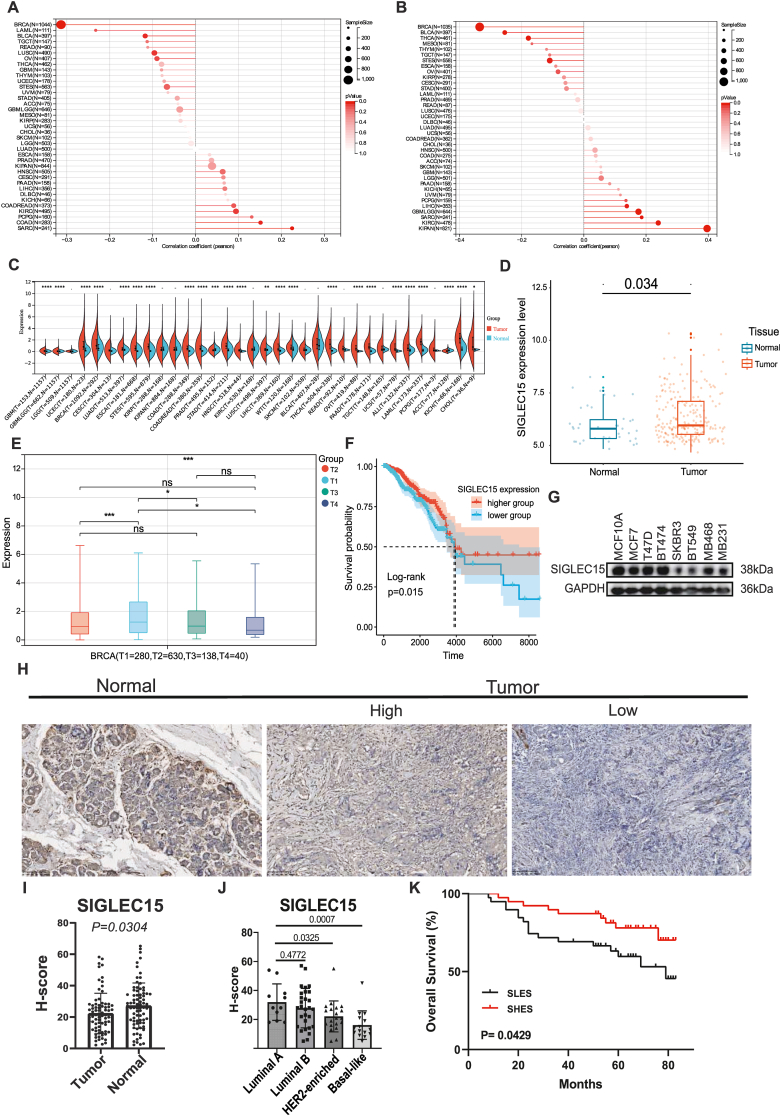
Distribution of *SIGLEC15* in BRCA patients. **(A)** The correlation between *SIGLEC15* and homology-directed repair (HRD) of pan-cancer. **(B)** The correlation between *SIGLEC15* and loss of heterozygosity (LOH) of pan-cancer. **(C)** The expression of *SIGLEC15* in pan-cancer between tumor tissue and normal tissue in datasets TCGA-BRCA. **(D)** The expression of *SIGLEC15* between tumor tissue and normal tissue in data sets GSE162228 and GSE42568. **(E)** The *SIGLEC15* expression level in BRCA at different T stages. **(F)** Kaplan–Meier plot for patients with BRCA grouped by the median *SIGLEC15* expression level in TCGA-BRCA. **(G)** Western blot of *SIGLEC15* in eight BRCA cell lines. **(H)** The immunohistochemical staining of tumor tissue of BRCA patients for *SIGLEC15*. **(I)** Quantitative analysis of immunohistochemical staining between primary tumor tissue and normal breast tissue. **(J)** Quantitative analysis of immunohistochemical staining of primary tumor tissue from patients in the PAM50 molecular subtypes including Luminal A, Luminal B, Her2-enriched and Basal-like. **(K)** Kaplan–Meier plot for patients with BRCA grouped by the median *SIGLEC15* expression level from Chongqing Medical University.

To estimate the potential of *SIGLEC15* as a cancer therapeutic target, we examined the homology-directed repair (HRD) and loss of heterozygosity (LOH) of *SIGLEC15* in multiple cancers, which are strongly associated with sensitivity to platinum-based drugs and poly ADP-ribose polymerase (PARP) inhibitors (Fig. 1A, B). The correlation coefficient of *SIGLEC15* in BRCA indicated that patients with higher *SIGLEC15* expression levels have lower sensitivity to platinum and PARP inhibitors, demonstrating the potential of *SIGLEC15* as a therapeutic target.

Using datasets from TCGA and GTEx, we found clear differences in the expression level of *SIGLEC15* between various types of tumor tissues and their corresponding normal tissues, including BRCA (Fig. 1C). We then focused on BRCA and found that the *SIGLEC15* expression level in BRCA tumors was higher than that in normal tissues. To confirm this result, we examined the expression level of *SIGLEC15* using two GEO datasets (GSE162228 and GSE42568) (Fig. 1D). The results were similar to those calculated with data from TCGA and GTEx.

Next, we found significant differences between the T1 and T2 stages, as well as between the T1 and T3 stages, in the tumor tissues (Fig. 1E). A higher *SIGLEC15* expression level in T1 stage BRCA patients suggested that *SIGLEC15* may be a marker for early stage BRCA. Then we explored the role of *SIGLEC15* in tumor tissue. The survival rates of patients with higher *SIGLEC15* expression level (SHES) were significantly higher than those with lower expression level (SLES) (Fig. 1F), indicating that *SIGLEC15* is a protective factor for BRCA patients. A higher *SIGLEC15* expression level was associated with increased survival in these BRCA patients.

Then we studied the expression levels of *SIGLEC15* in BRCA cell lines (Fig. 1G). We observed that *SIGLEC15* expression was significantly higher in normal breast epithelial cells (MCF10A) and estrogen receptor-positive breast cancer cell lines, but markedly lower in estrogen receptor-negative breast cancer cell lines. These findings provide valuable reference points for our subsequent experiments. To validate this result, we conducted immunohistochemical staining of *SIGLEC15* in paired primary tumor and adjacent non-cancerous tissues from 64 breast cancer patients (Fig. 1H). Quantitative analysis revealed significantly higher *SIGLEC15* expression in normal adjacent tissues than in tumor tissues (Fig. 1I). Furthermore, when patients were stratified by the PAM50 molecular subtypes, luminal-type BRCA exhibited markedly elevated *SIGLEC15* protein levels relative to the HER2-enriched and basal-like subtypes (Fig. 1J). Survival analysis showed that the SHES cohort has a higher five-year survival rate (Fig. 1K). These results indicate that low *SIGLEC15* expression predicts higher malignancy and poor outcomes.

### *SIGLEC15* affects the TME of BRCA

**Figure 2:**
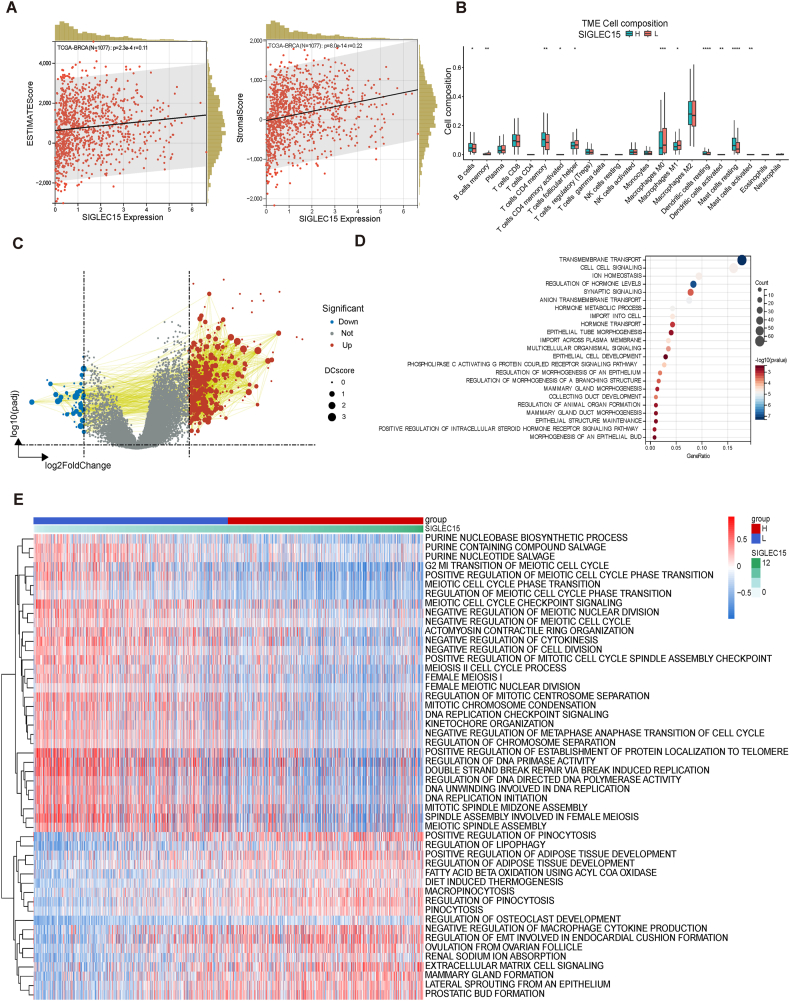
The immune cell landscape with different *SIGLEC15* expression level and its predicted functions depend on TCGA-BRCA. **(A)** Estimate score of *SIGLEC15* in TCGA-BRCA. (left) Estimate score. (right) Stromal score. **(B)** Immune infiltration analysis of datasets TCGA-BRCA grouped by the median *SIGLEC15* expression level calculated via Cybersort. **(C)** Different analysis between different *SIGLEC15* expression level groups in TCGA-BRCA grouped by the median *SIGLEC15* expression level. **(D)** Pathway enrichment of the different genes resulted from different analyses depends on the REACTOME database and KEGG database. **(E)** Gene Set Variation Analysis (GSVA) of different *SIGLEC15* expression level groups in TCGA-BRCA grouped by the median *SIGLEC15* expression level depends on the datasets GO_BP, GO_CC, and GOMF.

To investigate whether *SIGLEC15* influences the TME of BRCA, we evaluated the relationship between the *SIGLEC15* expression level and tumor tissue cells using ESTIMATEScores. Positive correlations were observed between the *SIGLEC15* expression level and the presence of immune cells and stromal cells in BRCA samples (Fig. 2A).

We compared the cell composition between the SHES and SLES groups using CIBERSORT analysis. This revealed that as *SIGLEC15* expression increases, the number of M0 and M1 macrophages decrease, while dendritic cells increase, indicating enhanced anti-tumor immune surveillance (Fig. 2B). Additionally, naïve B cells and mast cells increased in the SHES group, suggesting the activation of humoral immunity and inflammation in the TME. *SIGLEC15* may suppress cellular immunity.

Next, we performed differential analysis to identify differences between the SHES and SLES. We found 71 down-regulated genes and 444 up-regulated genes between SHES and SLES (Fig. 2C). Among these genes, several are known to be involved in important biological processes of BRCA, such as *PIP, ANKRD30A, LINC00993, and TFF1*. Pathway enrichment analysis using the REACTOME and KEGG databases revealed that the differentially expressed genes are involved in cell transportation, epithelial cell development, and cell structure changes (Fig. 2D).

GSVA of groups with different *SIGLEC15* expression levels indicated that, in the SHES group, cell division affections and energy metabolism functions were down-regulated, while functions like lipid metabolism and cell metaplasia were up-regulated (Fig. 2E). This suggests that with higher *SIGLEC15* expression, humoral immunity may be activated and cell metaplasia may occur alongside changes in energy metabolism.

### *SIGLEC15* is associated with different TME compositions and proportions of malignant cells

**Figure 3:**
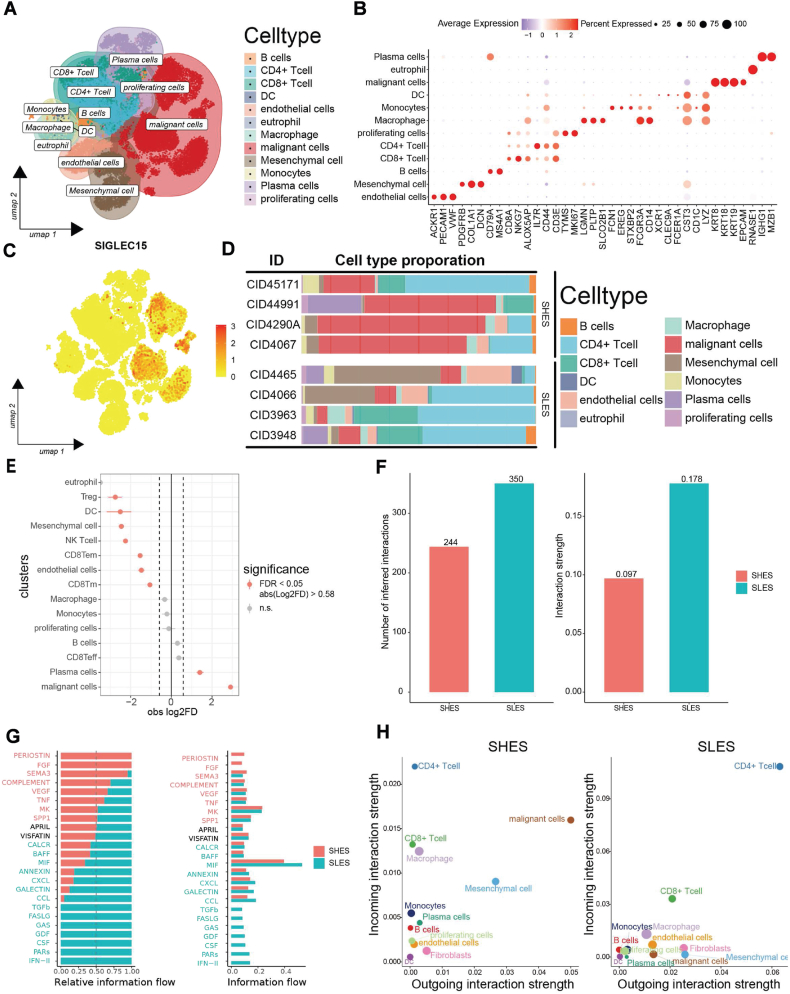
Single-cell analysis revealed the functions of *SIGLEC15* in the TME. **(A)** Uniform manifold approximation and projection (umap) of BRCA cells in the GSE176078 dataset. **(B)** Markers for identifying cell types in single-cell datasets. **(C)** Feature plot of *SIGLEC15* in BRCA tissue cells. **(D)** The cell type proportions of every sample grouped by the median *SIGLEC15* expression level. **(E)** The difference test of cell proportions of cell type in different groups depends on R-package scProportionTest. **(F)** The number and strength of interactions in the primary tumor TME. **(G)** Relative information of interactions in the primary tumor TME. **(H)** Difference of the incoming and outgoing interactions of the cells in SHES and SLES.

Our objective was to determine the specific cell types in which SIGLEC15 is predominantly expressed. Using the single-cell RNA sequencing dataset GSE176078, we found that SIGLEC15 is primarily expressed in epithelial cells (Fig. 3A). The marker genes for each cell type are presented in Figure 3B, which corroborates that SIGLEC15 is chiefly expressed in malignant cells (Fig. 3C). Furthermore, the proportions of each cell type were compared between the two groups (Fig. 3D). Subsequently, we analyzed the subtypes of T cells (Fig. S1A, S1B).

To better understand the composition of the TME, we compared all cell types from SHES with their counterparts in SLES (Fig. 3E). The proportions of malignant cells were higher in SHES while the proportions of Tregs, mesenchymal cells, NK T cells, and CD8 Tems were higher in SLES. The significant difference in immune cell and malignant cell proportions between SHES and SLES led us to focus on the TME. Using CellChat, we observed a lower interaction strength and fewer interactions in SHES (Fig. 3F).

The higher signaling pathways in SHES included PERIOSTIN, FGF, SEMA3, COMPLEMENT, VEGF, TNF, MK, and SPP1, while those in SLES included CALCR, BAFF, MIF, ANNEXIN, CXCL, GALECTIN, CCL, TGFb, FASLG, GAS, GDF, CSF, PARS, and IFN-2 (Fig. 3G). We observed notable difference in cell–cell interactions between SHES and SLES, particularly among CD4^+^ T cells, malignant cells, and mesenchymal cells (Fig. 3H).

### SIGLEC15 expression impairs T cell infiltration in the breast cancer TME

**Figure 4:**
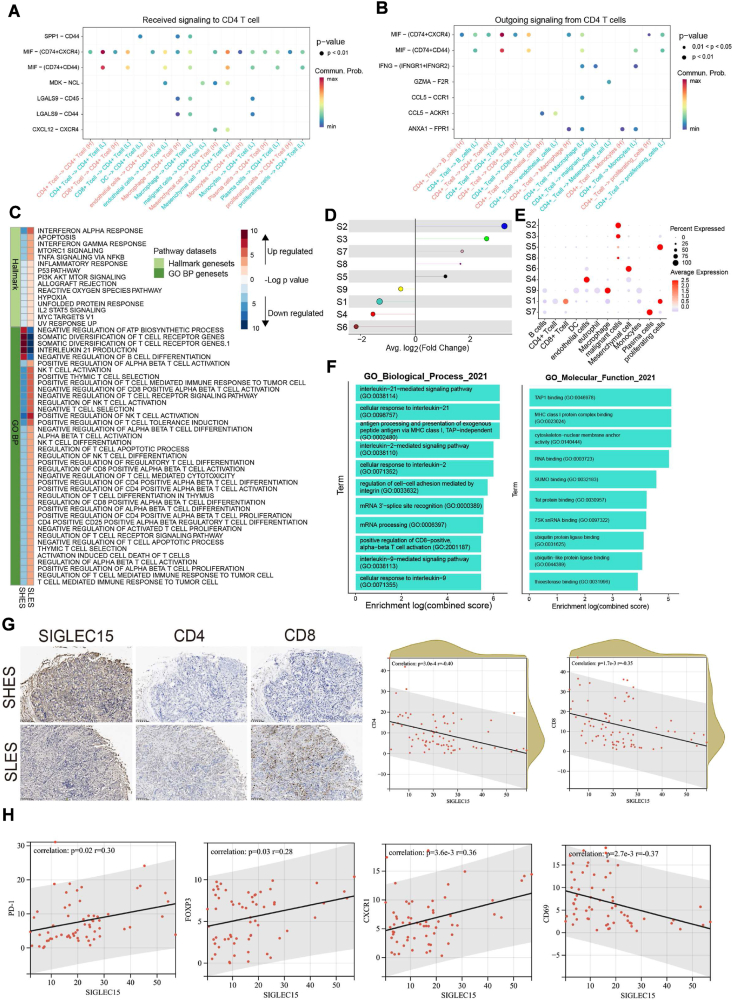
Analysis of the primary BRCA TME showed the significant T cell inhibition in SHES. **(A)** Significantly different ligand-target signaling of CD4^+^ T cells received in the TME. **(B)** Significantly different ligand-target signaling of CD4^+^ T cells output in the TME. **(C)** Pathway score test analysis of CD4^+^ T cells between SHES and SLES. **(D)** Relationship of differential gene regulatory network between SHES and SLES by hdWGCNA. **(E)** Relationship of differential gene regulatory network in kinds of cell phenotypes by hdWGCNA. **(F)** The pathways enriched by S1 depend on the datasets GO_BP and GO_CC. **(G)** Analysis of the relationship among CD4, CD8 and *SIGLEC15* in the immunohistochemical results of 64 BRCA patients. **(H)** Analysis of the relationship among PD1, FOXP3, CD69, CXCR1 and *SIGLEC15* in the immunohistochemical results of 64 BRCA patients.

Then we focused on CD4^+^ T cells. We focused on the role of CD4^+^ T cells, which, in the SLES group, demonstrated increased receipt and secretion of macrophage migration inhibitory factor (MIF) from and to other cells, respectively. This phenomenon is recognized for its role in suppressing anti-tumor immunity (Fig. 4A, B). CD4^+^ T cells were particularly noteworthy due to their markedly distinct interactions and proportions between the SLES and SHES groups. Pathway score analysis indicated that CD4^+^ T cells in the SLES group exhibits increased signaling pathways, including those involving IFN-γ, IFN-α, and IL-2. Conversely, pathways related to T cell activation and the T cell-mediated tumor response were significantly down-regulated in the SHES group (Fig. 4C). This suggests a potentially hazardous factor that could facilitate tumor immune responses and metastasis. Despite the lower proportion of malignant cells observed in SLES, we propose that the active anti-tumor immunity in this group effectively eradicated most malignant cells through targeted immune responses. Nonetheless, MIF present in the TME might confer protection to the residual malignant cells, enabling them to evade immune-mediated elimination.

We employed hdWGCNA to conduct an in-depth investigation of the dynamic functional modules within the TME. Using this analysis, we found nine modules that are correlated with *SIGLEC15* expression (Fig. S1D). Positive correlations were observed in modules S2, S3, S8, S7, and S5, while the remaining modules showed negative correlations in SHES (Fig. 4D). These modules exhibited inter-module correlations, and each module could be mapped to specific cell type clusters (Fig. 4E). Our findings indicated that the S1 gene module exhibited elevated expression levels in CD8^+^ T cells derived from SLES (Fig. 4D, E). The pathways enriched in the S1 module genes suggest the activation of CD8^+^ T cells, including IL-21, IL-9, and IL-2 signaling, and T cell receptor complexes (Fig. 4F). In the context of reduced SIGLEC15 expression, T cells within the TME are activated and concurrently subjected to increased MIF stimulation. Notably, MIF is a critical factor associated with malignant cell metastasis, as it impedes the cytotoxic activity of CD4^+^ and CD8^+^ T cells against local tumor cells.

To validate the findings of our bioinformatic analysis, we conducted slice staining to examine the conditions within the population (Fig. 4G). In the tumor tissues of breast cancer patients, SIGLEC15 expression was found to be negatively correlated with CD4 and CD8 expression levels. Furthermore, increased expression of SIGLEC15 was associated with higher levels of PD-1, FOXP3 and, CXCR1, and lower levels of CD69 in tumor tissues (Fig. 4H; Fig. S1E). These observations further substantiate the role of SIGLEC15 in suppressing T cell function within the TME of breast cancer.

### *SIGLEC15* expression suppresses EMT and metastatic capacity in breast cancer

**Figure 5:**
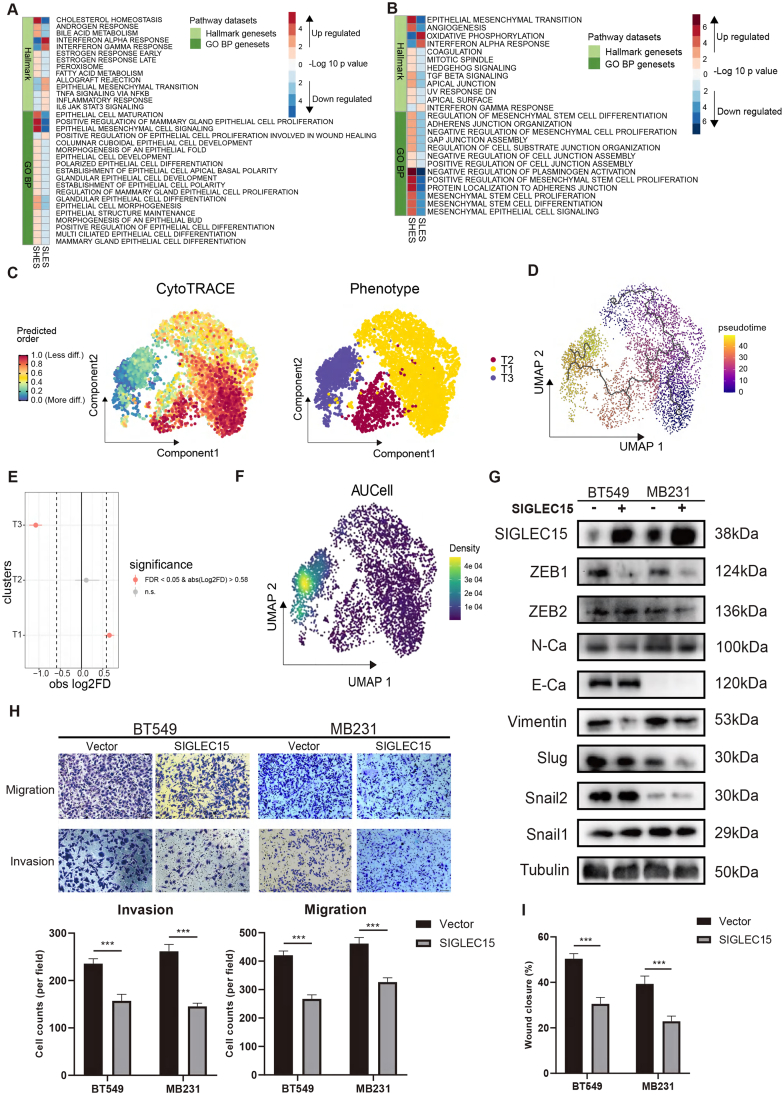
SIGLEC15 expression suppresses EMT and metastatic capacity in breast cancer. **(A)** Pathway score test analysis for malignant cells between SHES and SLES. **(B)** Pathway score test analysis for mesenchymal cells between SHES and SLES. **(C)** Cytometry Trace (Cytotrace) analysis showed the differential power of cells in BRCA bone metastasis from the GSE190772 dataset. (left) Differential power of the cells (right). Subcluster of malignant cells in BRCA bone metastasis. **(D)** Differential trajectories of malignant cells calculated via monocle3. **(E)** The difference test of cell proportions of malignant cell subclusters in different groups depend on R-package scProportionTest. **(F)** The EMT pathway score test analysis for malignant cell subclusters in BRCA bone metastasis from the GSE190772 dataset calculated by AUCell. **(G)** Western blot analysis detected changes in EMT-related protein levels in *SIGLEC15*-overexpressing BT549 and MB231 cells. **(H)** Transwell assays examined changes in the migration and invasion of *SIGLEC15*-overexpressing BT549 and MB231 cells. Quantitative analysis of the results were performed. **(I)** Quantitative analysis for the wound-healing test results. ∗*P* ≤ 0.05. ∗∗*P* ≤ 0.01. ∗∗∗*P* ≤ 0.001.

In our analysis, the SHES subgroup exhibited a significantly higher proportion of malignant cells and a lower proportion of mesenchymal cells, accompanied by suppression of the EMT pathway. Mechanistically, SIGLEC15 expression was found to inhibit the EMT pathway in breast cancer cells, thereby impeding the transdifferentiation of malignant cells into mesenchymal-like phenotypes. Pathway score analysis of malignant cells supports this hypothesis (Fig. 5A), showing that the EMT pathway was down-regulated in SHES. This is accompanied by a down-regulation of the allograft rejection pathway, along with significant activation of pathways related to epithelial cell morphological maturity and maintenance. Conversely, in mesenchymal cells, the up-regulated EMT pathway, along with improvements in the apical surface and apical junction, as well as the up-regulation of mesenchymal cell development pathways in SHES, highlights the active functionality of mesenchymal cells, potentially linked to the tight connections between these cells^12^^,^^39^ (Fig. 5B).

Based on these findings, we propose that malignant cells exhibit distinct EMT statuses between the two groups, which might contribute to differences in tumor metastasis. To test this hypothesis, we used CytoTRACE to estimate the differentiation status of malignant cells in BRCA bone metastasis epithelial cells (Fig. 5C; Fig. S2A, S2B). Malignant cells were divided into three subclusters (T1, T2, and T3), where T1 and T2 displayed higher differentiation potential than T3. A differentiation trajectory calculated using monocle3 showed that T1 and T2 clusters can differentiate into T3 cluster (Fig. 5D). Notably, SHES showed a significantly higher proportion of T1 cells and a lower proportion of T3 cells compared to SLES (Fig. 5E). We used AUCell to analyze EMT pathway scores in SHES and SLES (Fig. 5F). Compared to T1 and T2, T3 showed up-regulation in the EMT pathway.

To prove this finding, we generated SIGLEC15-overexpressing BT549 and MB231 breast cancer cell lines and validated the protein expression levels of EMT pathway-related molecules (Fig. 5G). The results showed that the overexpression of SIGLEC15 significantly reduced the protein expression levels of ZEB1, N-cadherin, and vimentin. in MB231 and BT549.^40^^,^^41^ These findings indicate that the EMT program is suppressed, suggesting a potential impact on the metastatic capacity of breast cancer cells.^42^ To further validate the role of SIGLEC15 in EMT and metastasis, we performed wound healing wound healing and Transwell assays. In SIGLEC15-overexpressing BT549 and MB231 cells, both the migration and invasion capacities were significantly decreased compared to control cells (Fig. 5H, I; Fig. S2C). Conversely, SIGLEC15-knockdown MB231 cells exhibited enhanced migration and invasion abilities, accompanied by increased protein levels of the EMT master regulator ZEB1 and the mesenchymal marker N-cadherin (Fig. S2D–S2H). These function experiments collectively demonstrated that SIGLEC15 suppresses breast cancer cell EMT progression and metastatic capacity.

### *SIGLEC15* inhibits the EMT process by down-regulating *ZEB1* expression

**Figure 6:**
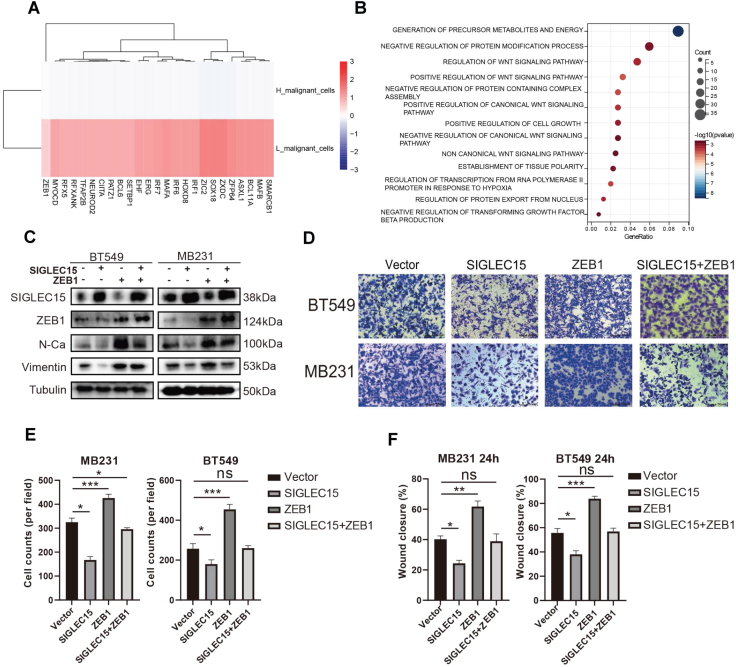
*SIGLEC15* inhibits the EMT process by down-regulating ZEB1 expression. **(A)** Transcription factors analysis of malignant cells from primary BRCA tumors calculated by decoupleR, including the transcription factors with differences. **(B)** The pathways enriched by the S3 module. **(C)** WB analysis was conducted to evaluate the effects of ZEB1 overexpression on rescuing *SIGLEC15*-overexpressing inhibition of key proteins in the EMT pathway in BT549 and MB231 cells. **(D)** Transwell migration assay results demonstrated that overexpression of ZEB1 rescued the inhibitory effect of SIGLEC15 overexpression on the migratory ability of BT549 and MB231 cells. **(E)** Quantitative analysis for the Transwell test results. **(F)** Quantitative analysis for the wound-healing test results. ∗*P* ≤ 0.05. ∗∗*P* ≤ 0.01. ∗∗∗*P* ≤ 0.001.

In our analysis, the SHES subgroup exhibited a significantly higher proportion of malignant cells and a lower proportion of mesenchymal cells, accompanied by suppression of the EMT pathway. Mechanistically, SIGLEC15 expression was found to inhibit the EMT pathway in breast cancer cells, thereby impeding the transdifferentiation of malignant cells into mesenchymal-like phenotypes. The relationship between SIGLEC15 and EMT remains unclear. To address this question, we used decoupleR in malignant cells to identify significantly altered transcription factors (Fig. 6A). ZEB1, which can promote the biological processes of EMT,^43^ was found to be highly expressed in SLES. Due to the down-regulated ZEB1 in the SIGLEC15 overexpressing cells, we put ZEB1 was the downstream factor of SIGLEC15.

Next, we performed hdWGCNA on the scRNA-seq samples to identify gene modules associated with the SIGLEC15 expression levels. Among these, modules S2, S3, and S8 displayed greater correlations in malignant cells where SIGLEC15 was expressed. Functional enrichment analysis using the GOBP pathway of module S3 revealed significant pathways related to ZEB activity, such as the TGF-beta and Wnt3 signaling pathways (Fig. 6B).

In order to confirm the relationship between SIGLEC15 and ZEB1, we constructed a ZEB1 overexpression plasmid. After ZEB1 overexpression, the EMT pathway of BT549 and MB231 breast cancer cells was up-regulated, as shown by the increase in N-cadherin and Vimentin expression. And ZEB1 rescued the inhibition of the EMT pathway caused by SIGLEC15 overexpression (Fig. 6C). The same result was also found in the *in vitro* experiment. ZEB1 overexpression rescued the ability of SIGLEC15 to inhibit TNBC cell migration and invasion (Fig. 6D–F; Fig. S3). SIGLEC15 inhibits the EMT pathway and modulates the biological behavior of breast cancer cells by regulating ZEB1 expression.

### *SIGLEC15* regulates TNBC proliferation and develops different treatment strategies

**Figure 7:**
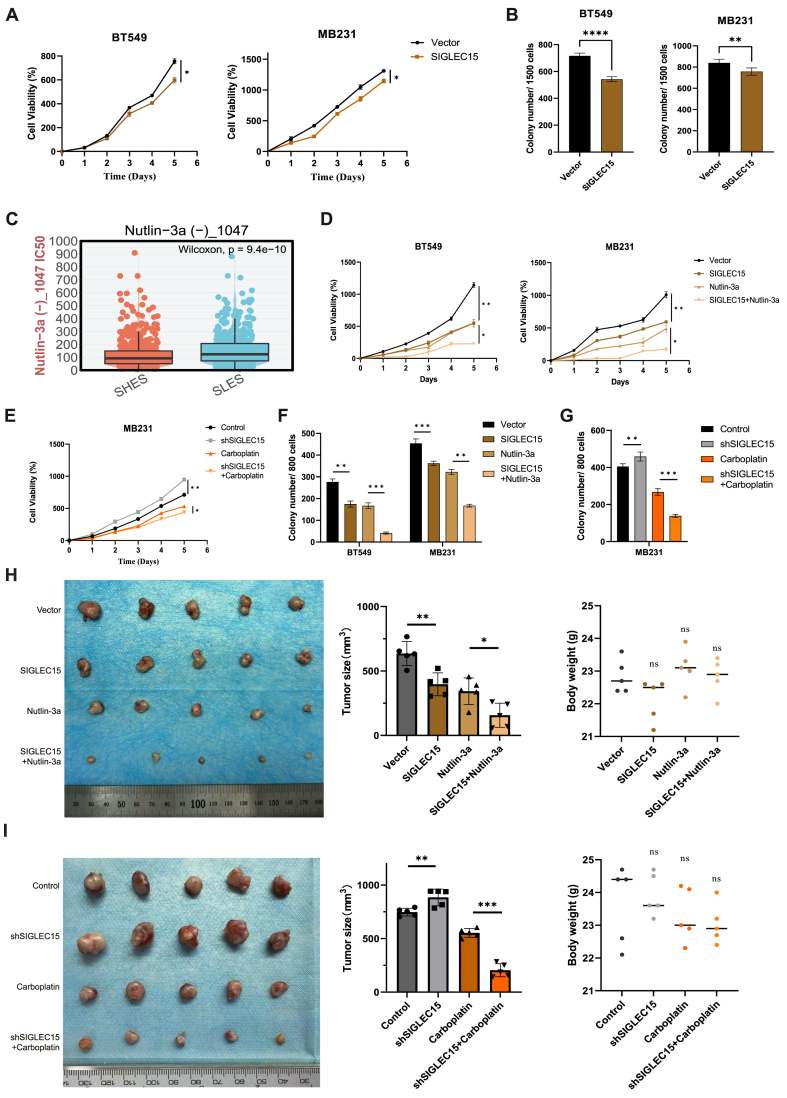
*In vivo* and *in vitro* experiments validated the effect of *SIGLEC15* on breast cancer proliferation and explored therapeutic strategies based on its expression level. **(A)** Comparison of the cell proliferation between overexpression and control group of the BT549 and MB231 cells using the CCK8 method. **(B)** Quantitative analysis of the colony formation assay in BT549 and MB231. **(C)** SHES and SLES in TCGA-BRCA were analyzed for differences in the drug resistance of Nutlin-3a through IC50 analysis. **(D, E)** CCK-8 assay and colony formation assay were used to evaluate the therapeutic effect of Nutlin-3a on SIGLEC15-overexpressing BT549 and MB231 cells. **(F, G)** CCK-8 and colony formation assays verified the inhibitory effect of carboplatin on SIGLEC15-knockdown MB231 cells. **(H)** MB231 cells (vector and SIGLEC15 groups) were subcutaneously injected into the left mammary fat pads of 4-week-old female BALB/c nude mice. Beginning on day 10 post-injection, the mice received daily intraperitoneal injections of either Nutlin-3a (5 mg/kg) or physiological saline (control). Tumor size was measured after the mice were euthanized. Representative tumor images were captured for each group (*n* = 5 mice/group). Statistical analysis of the final tumor size and mouse body weight was performed. **(I)** MB231 cells (shCrtl and shSIGLEC15 groups) were subcutaneously injected into the left mammary fat pads of 4-week-old female BALB/c nude mice. Beginning on day 10 post-injection, the mice received intraperitoneal injections of either carboplatin (60 mg/kg) or physiological saline (control) each week. Tumor size was measured every 3 days for 30 days after the mice were euthanized. Representative tumor images were captured for each group (*n* = 5 mice/group). Statistical analysis of the final tumor size and mouse body weight was performed. “ns” represents not significant, ∗*P* ≤ 0.05. ∗∗*P* ≤ 0.01. ∗∗∗*P* ≤ 0.001.

Previously, we observed that high expression of *SIGLE*C15 confers resistance to platinum-based drugs and is associated with reduced immune cell infiltration, which may contribute to the diminished efficacy of immunotherapy. Infinite cell division and proliferation are defining characteristics of this biological context. We first investigated the alterations in the proliferation capacities of MB231 and BT549 cells following SIGLEC15 overexpression. The data revealed that SIGLEC15 overexpression notably reduced both the cell plate clone formation efficiency and cellular viability. Conversely, SIGLEC15 knockdown resulted in a significant increase in the proliferation ability of MB231 cells (Fig. 7A, B; Fig. S4A–S4C). Additionally, our analysis of cell cycle-associated proteins demonstrated that a negative correlation between the protein expression of SIGLEC15 and that of Cyclin D1 (Fig. S4D, S4E). Then we utilized the GDSC database for breast cancer to screen for potential drugs targeting SIGLEC15-overexpressing breast cancer cells (Fig. 7C). Our findings revealed that Nutlin-3a exhibited the highest sensitivity among the candidates (Table S1). As an effective MDM2 inhibitor, Nutlin-3a inhibits the interaction between MDM2 and p53, stabilizes the p53 protein, and induces cellular autophagy and apoptosis. Therefore, we employed plate cloning assays and CCK8 proliferation assays to assess the impact of the drugs on the proliferation of SIGLEC15-overexpressing breast cancer cells. The results demonstrated that Nutlin-3a exerted a potent inhibitory effect on SIGLEC15-overexpressing cell lines (Fig. 7D, E). In contrast, carboplatin significantly suppressed the proliferation of SIGLEC15-knockdown MB231 cells (Fig. 7F, G; Fig. S4G).

**Figure 8:**
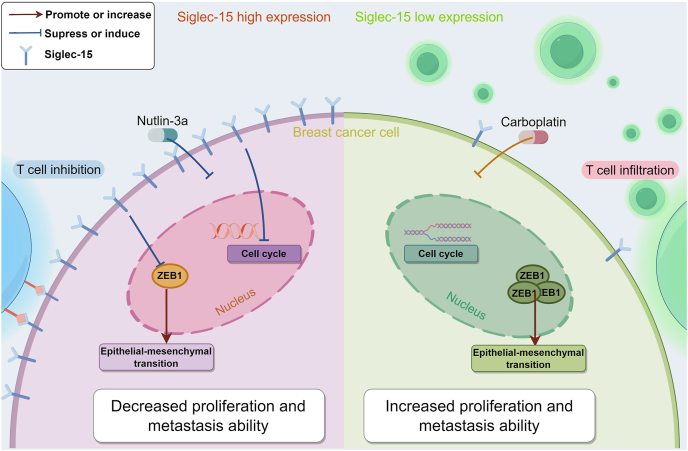
Mechanism of the heterogeneity of SIGLEC15 expression in regulating breast cancer cell–microenvironment interactions and targeted therapeutic strategies.

Furthermore, we validated these findings *in vivo*, and the results were consistent with those from *in vitro* experiments, demonstrating that tumors with higher SIGLEC15 expression levels were significantly smaller than those in the control group. Treatment with Nutlin-3a further inhibited the growth of high-SIGLEC15-expressing tumors (Fig. 7H). Additionally, we conducted *in vivo* tumorigenesis experiments using MB231 cells with SIGLEC15 knockdown. The results showed that the *in vivo* tumorigenic volume of the SIGLEC15-knockdown MB231 cells was greater than that of the control group, and carboplatin exhibited a potent inhibitory effect on low-SIGLEC15-expressing tumors (Fig. 7I). Through integrated analysis of database data, clinical samples, and *in vivo*/*in vitro* experiments, we comprehensively elucidated the role of SIGLEC15 in breast cancer, particularly in triple-negative breast cancer (Fig. 8).

## Discussion

Our study analyzed the differences in the TME of patients with varying *SIGLEC15* expression levels using both bulk RNA-seq and scRNA-seq data. Based on the better prognosis in the SHES group, we considered *SIGLEC15* a suppressor of BRCA. Single-cell analysis revealed that SHES is characterized by a higher proportion of malignant cells and a suppressed T cell immune landscape. Previous studies have demonstrated that higher *SIGLEC15* expression in macrophages inhibits T cell activity and promotes the accumulation of M2 pro-tumor macrophages.^44^

Compared with the SHES and SLES pathway differences revealed by RNA sequencing, changes in mammary gland form and epithelial cell morphology were up-regulated in SHES compared to those with lower cell division course. Higher mammary gland morphology and epithelial cell development could explain the mammary gland cells with higher expression of *SIGLEC15* in early primary BRCA tissue. A previous study reported that *SIGLEC15* is related to attenuation of the immune response by interacting with Siglec receptors in natural killer cells, dendritic cells, and monocytes.^45^ Interestingly, overexpression of the *SIGLEC15* protein in colorectal tumor cells is associated with advanced TNM stage and predicts fewer tumor-infiltrating lymphocytes.^46^ Similarly, the significant changes in cell division and the reduced infiltration of macrophages and B cells in SHES suggest that *SIGLEC15* may impact not only lymphocytes but also other cell types. An analysis of the scRNA-seq data revealed that T cell inhibition was observed in the higher *SIGLEC15* expression group, suggesting the possibility that *SIGLEC15* may play similar roles in T cells as previously reported, both in epithelial cells and in macrophages. Consistently, our IHC analysis of patient samples demonstrated a negative correlation between *SIGLEC15* expression and the expression levels of CD4 and CD8 in BRCA tissue.

In the TME, we observed a significantly lower number and weaker strength of cell-to-cell interactions in SHES. Specifically, the lower incoming and outgoing interactions of CD4^+^ T cell in SHES indicate that CD4^+^ T cells are significantly suppressed. Pathway enrichment analysis of CD4^+^ T cells in SHES and SLES further confirmed the suppression of CD4^+^ T cells in SHES. In addition, the up-regulation of the somatic diversification of T cell receptor genes in SHES suggests a possible reason for this suppression. Our analysis of cell ligand–receptor interactions showed that the level of the pro-tumor factor MIF^47^ in CD4^+^ T cells was lower in SHES than in SLES. Furthermore, the expression of hub genes related to the IL-21 signaling pathway in CD8^+^ T cell was down-regulated in SHES, which was associated with reduced CD8^+^ T cell activation.^49^ These findings provide new insights into immune regulation and offer a novel perspective on immune control mechanisms in the TME.

The EMT pathway was down-regulated in tumor cells from SHES, similar to what was observed in patients from the TCGA-BRCA dataset. In contrast, tumor cells from SLES of BRCA bone metastasis had less differentiation ability. If *SIGLEC15* acts as an EMT inhibitor, it may facilitate metastatic colonization and tumor growth through mesenchymal–epithelial transition (MET).^48^ Furthermore, through bioinformatics analysis and our *in vitro* studies, we identified that *SIGLEC15* may influence the EMT pathway by modulating the expression of ZEB1, thereby affecting the invasion and migration capabilities of TNBC cells.

In addition, we observed that *SIGLEC15* exerts a significant impact on the cell cycle. Drawing on previous studies, we conducted *in vitro* experiments to investigate potential drug combinations.^50^, ^51^, ^52^ Our findings indicate that SIG exhibits a synergistic effect when combined with Nutlin-3a. In contrast, the administration of carboplatin to SLES resulted in tumor cell suppression. Moreover, the significantly suppressed tumor sizes in the SHES of mice suggest that the overall role of *SIGLEC15* in primary BRCA tumors is inhibitory, which is consistent with previous research.^44^ This finding implies that Nutlin-3a may be a more suitable therapeutic option for patients with high SIGLEC15 expression who do not respond to immunotherapy, whereas carboplatin may be more effective for patients with lower SIGLEC15 expression.

In conclusion, our study elucidates the dual role of SIGLEC15 within the TME of BRCA. Specifically, SIGLEC15 is implicated in the inhibition of T cell activity while concurrently mitigating tumor cell metastasis in BRCA. This bifunctional role indicates that therapeutic strategies could be more precisely tailored according to SIGLEC15 expression levels. For patients with TNBC exhibiting low SIGLEC15 expression, immune-based therapies or carboplatin may provide enhanced therapeutic efficacy. Conversely, Nutlin-3a may be more effective for patients with elevated SIGLEC15 expression levels.

## CRediT authorship contribution statement

**Zhaofu Tan:** Writing – original draft, Supervision, Software, Resources, Formal analysis, Conceptualization. **Hongbin Xin:** Writing – review & editing, Writing – original draft, Supervision, Methodology, Investigation, Data curation, Conceptualization. **Jian Chen:** Resources, Data curation. **Ming Lei:** Formal analysis. **Gang Tu:** Writing – review & editing, Funding acquisition. **Lingfeng Tang:** Writing – review & editing, Writing – original draft, Software, Resources, Methodology, Investigation, Funding acquisition, Conceptualization.

## Ethics declaration

This study received approval from the Ethics Committee of The First Affiliated Hospital of Chongqing Medical University (No. K2023-514). All animal experiments were approved by the Ethics Committee of Chongqing Medical University (IACUC-CQMU-2023-0267).

## Data availability

The RNA-sequencing data for the breast invasive carcinoma (BRCA) cohort utilized in this study are available in The Cancer Genome Atlas (TCGA) repository, accessible at https://portal.gdc.cancer.gov/. Further supporting datasets are available in the Gene Expression Omnibus (GEO) repository under accession numbers GSE162228, GSE42568, GSE176078, and GSE190772.

## Funding

This work was supported by The First Clinical College Clinical Medicine First-class Discipline Construction Project to Department of Breast and Thyroid Surgery to Lingfeng Tang (China) (No. CYYY-BSYJSCXXM-202322), The First Clinical College Clinical Medicine First-class Discipline Construction Project to Department of Breast and Thyroid Surgery to Zhaofu Tan (China) (No. CYYY-BSYJSCXXM-202333), and Innovative Research Group Project of the National Natural Science Foundation of China to Gang Tu (China) (No. 81372398).

## Conflict of interests

The authors declare that they have no competing interests.

## Appendix A Supplementary data

Multimedia component 1 **Figure S1***SIGLEC15* in the TME related to the immune suppression. **(A)** The UMAP of T cells' subcell types in the primary breast cancer TME from the GSE176078 datatset. **(B)** Markers used to identify T cells' subcell types. **(C)** The soft-thresholding power chosen for hdWGCNA is 7, which is based on the first instance of achieving a β-value of 0.8. **(D)** The gene modules in the TME of the primary BRCA tumors were calculated via the R-package hdWGCNA. Multimedia component 1

Multimedia component 2 **Figure S2***SIGLEC15* inhibits EMT biological processes in tumor cells. **(A)** The umap of the GSE190772 dataset. **(B)** Markers for identifying cell types in single-cell datasets. **(C)** The migration ability of BT549 and MB231 cells in the SIGLEC15-overexpressing group and the control group was compared via the wound healing test. **(D)** In the MB231 breast cancer cell lines, after transfection with blank control or knockdown lentivirus, the qPCR results showed a change in the level of *SIGLEC1*5 RNA. **(E)** After transfection with blank control or knockdown lentivirus, the WB results showed changes in the *SIGLEC15* protein in MB231 cells. **(F)** Western blot of SIGLEC15 and EMT-related proteins in the MB231 BRCA cells with SIGLEC15 knockdown. **(G)** (left) Transwell experiment of MB231 cells with SIGLEC15 knockdown. (right) Quantitative analysis for the Transwell test results. **(H)** (left) The scratch experiment of SIGLEC15-knockdown cell MB231. (right) Quantitative analysis for the scratch test results. ∗*P* ≤ 0.05. ∗∗*P* ≤ 0.01. ∗∗∗*P* ≤ 0.001. Multimedia component 2

Multimedia component 3 **Figure S3** The wound-healing assay revealed that *ZEB1* overexpression rescued the inhibitory effect of *SIGLEC15* on the migration of BT549 and MB231 cells. Multimedia component 3

Multimedia component 4 **Figure S4** Effects of *SIGLEC15* on the proliferation of TNBC cells and drug selection based on SIGLEC15 expression. **(A)** Comparison of the cell proliferation between the SIGLEC15 knockdown and control group of MB231 cell using the CCK8 method. **(B)** Comparison of the cell proliferation between the SIGLEC15 knockdown and control group of MB231 cell using the colony formation assay. Quantitative analysis for the colony formation assay results. **(C)** Comparison of the cell proliferation between the SIGLEC15 overexpression and control group of BT549 and MB231 breast cancer cells using the colony formation assay. **(D)** Western blot for cell cycle-related proteins in SIGLEC15-overexpression BT549 and MB231 cells. **(E)** Western blot for cell cycle-related proteins in SIGLEC15-knowdown MB231 cell. **(F)** Colony formation assay was used to evaluate the therapeutic effect of Nutlin-3a on SIGLEC15-overexpressing BT549 and MB231 cells. **(G)** Colony formation assays verified the inhibitory effect of carboplatin on SIGLEC15-knockdown MB231 cells. Multimedia component 4
