# Supplementary figures and images for: SIGLEC15 modulates the immunosuppressive microenvironment and suppresses malignant phenotypes in triple-negative breast cancer
Source: Genes Dis. 2025 Aug 9;13(1):101799. doi: 10.1016/j.gendis.2025.101799 (PMC12557572; doi:10.1016/j.gendis.2025.101799)

A

subcelltype

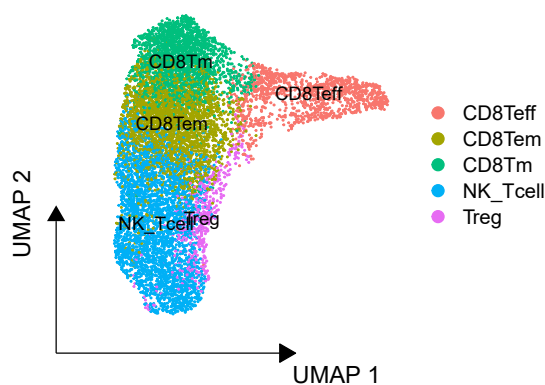

B

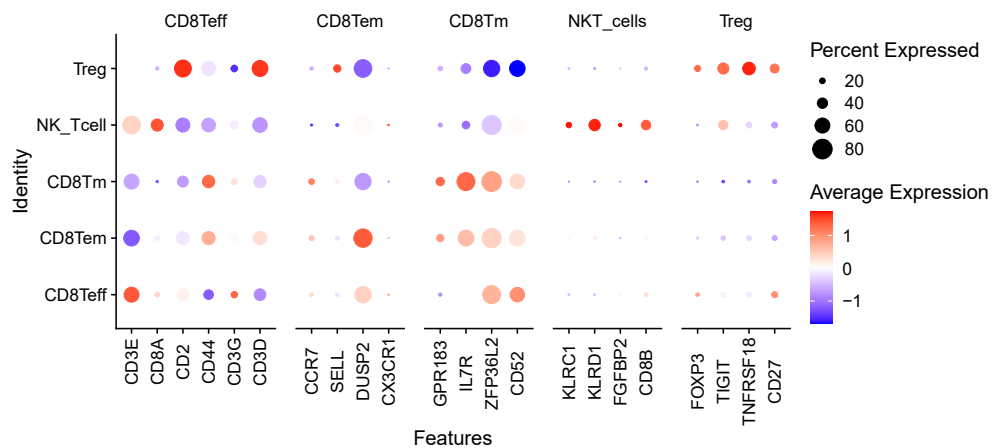

C

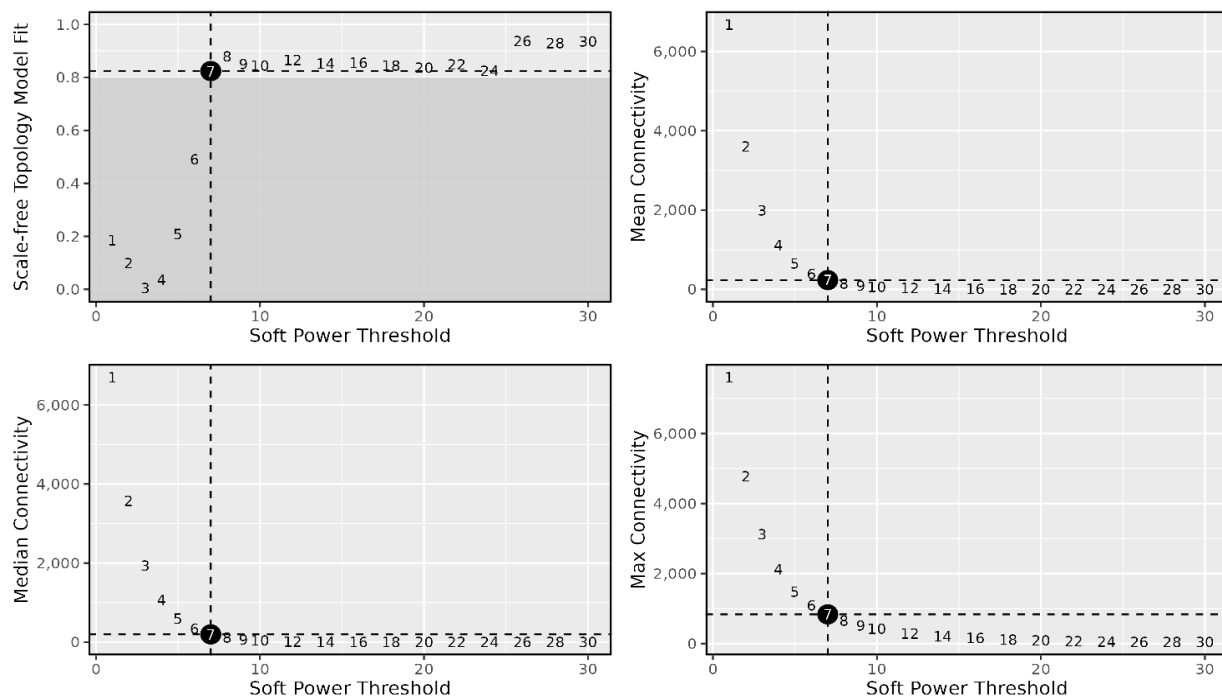

D

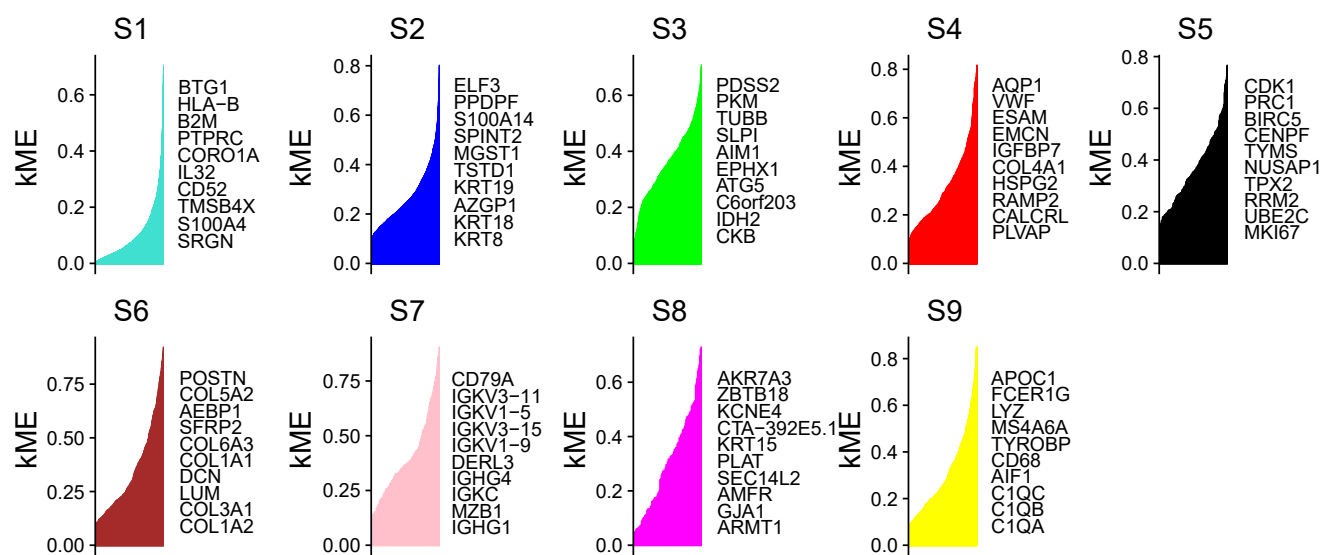

E

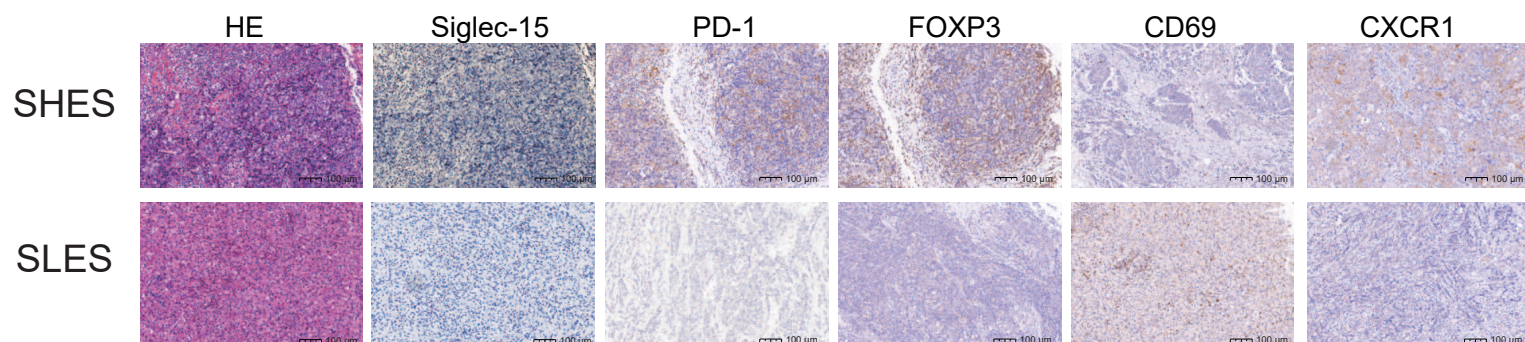

Supplement: Multimedia component 1 — Figure S1SIGLEC15 in the TME related to the immune suppression. (A) The UMAP of T cells' subcell types in the primary breast cancer TME from the GSE176078 datatset. (B) Markers used to identify T cells' subcell types. (C) The soft-thresholding power chosen for hdWGCNA is 7, which is based on the first instance of achieving a β-value of 0.8. (D) The gene modules in the TME of the primary BRCA tumors were calculated via the R-package hdWGCNA. [file mmc1.pdf]

**A**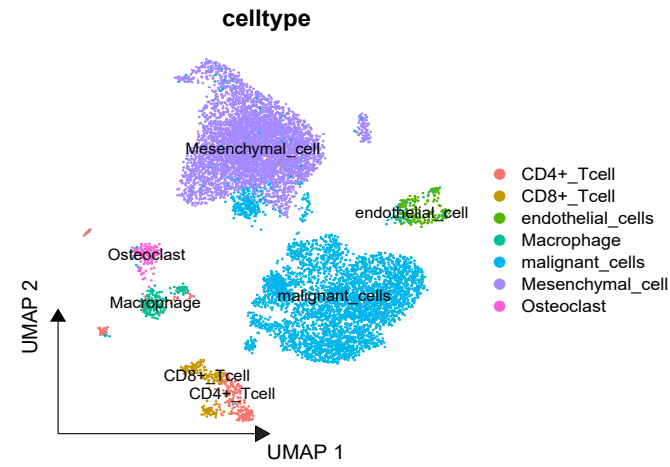**B**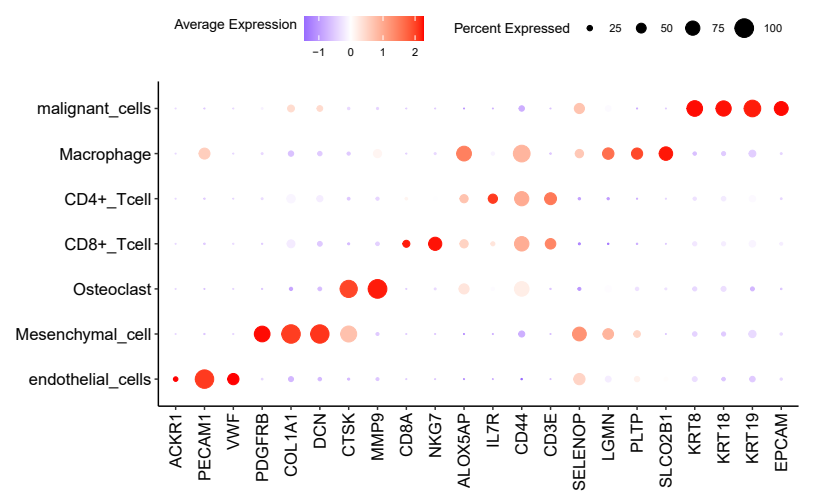**C**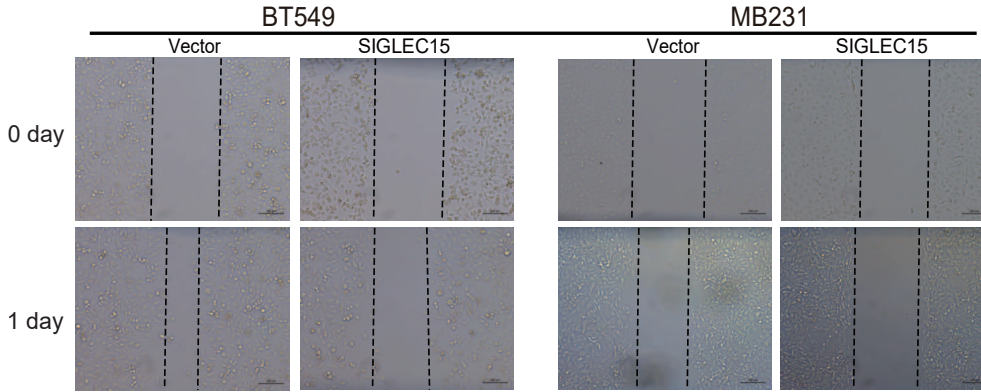**D**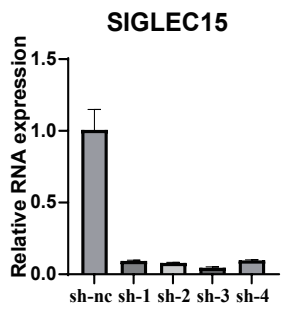**E**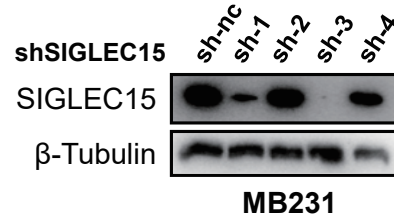**F**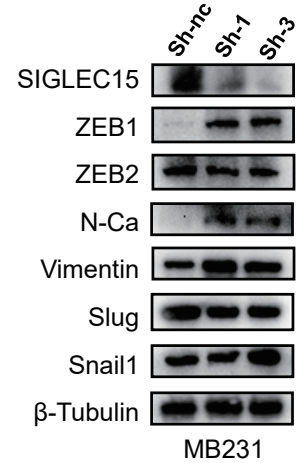**G**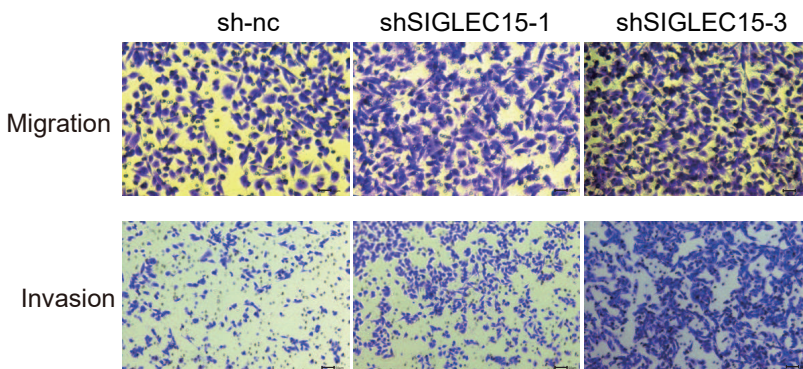**H**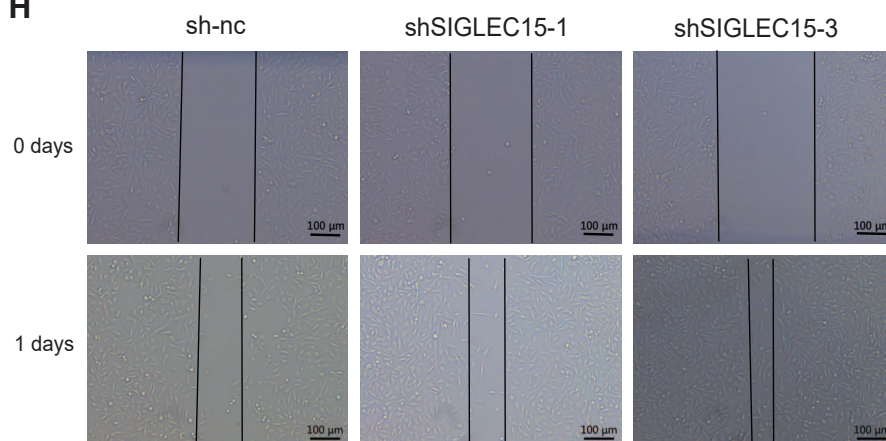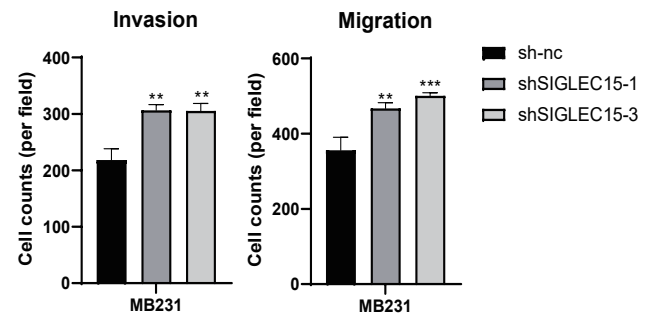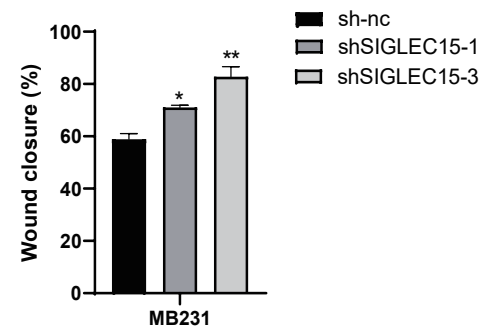

Supplement: Multimedia component 2 — Figure S2SIGLEC15 inhibits EMT biological processes in tumor cells. (A) The umap of the GSE190772 dataset. (B) Markers for identifying cell types in single-cell datasets. (C) The migration ability of BT549 and MB231 cells in the SIGLEC15-overexpressing group and the control group was compared via the wound healing test. (D) In the MB231 breast cancer cell lines, after transfection with blank control or knockdown lentivirus, the qPCR results showed a change in the level of SIGLEC15 RNA. (E) After transfection with blank control or knockdown lentivirus, the WB results showed changes in the SIGLEC15 protein in MB231 cells. (F) Western blot of SIGLEC15 and EMT-related proteins in the MB231 BRCA cells with SIGLEC15 knockdown. (G) (left) Transwell experiment of MB231 cells with SIGLEC15 knockdown. (right) Quantitative analysis for the Transwell test results. (H) (left) The scratch experiment of SIGLEC15-knockdown cell MB231. (right) Quantitative analysis for the scratch test results. ∗P ≤ 0.05. ∗∗P ≤ 0.01. ∗∗∗P ≤ 0.001. [file mmc2.pdf]

MB231

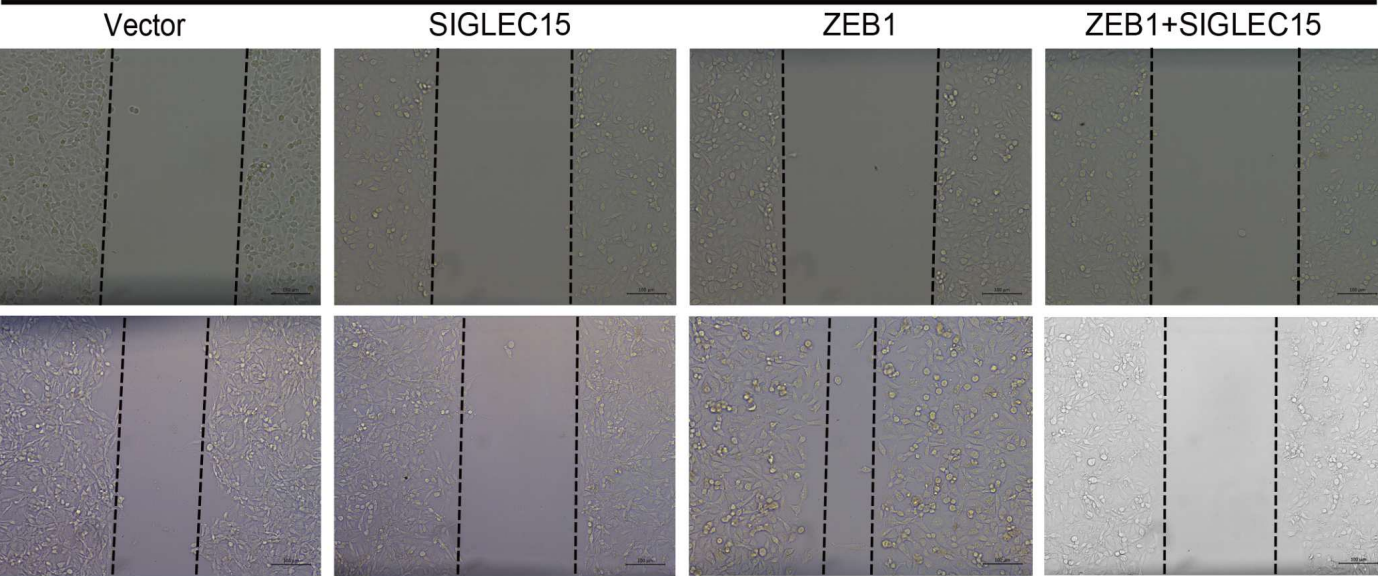

BT549

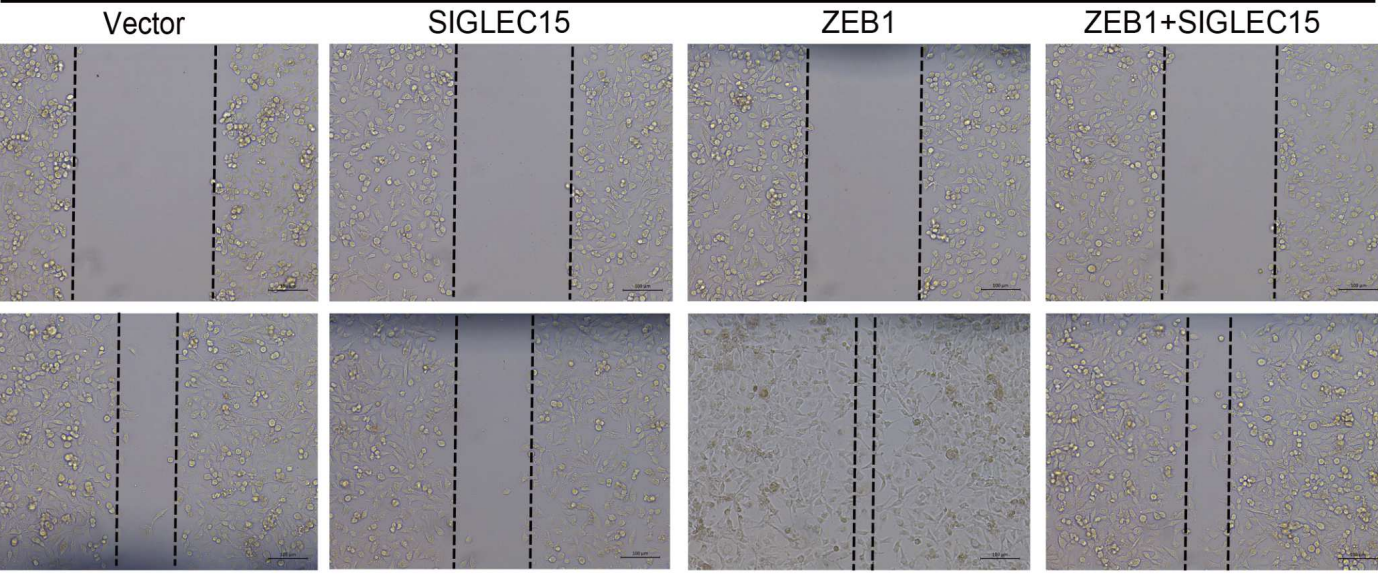

Supplement: Multimedia component 3 — Figure S3 The wound-healing assay revealed that ZEB1 overexpression rescued the inhibitory effect of SIGLEC15 on the migration of BT549 and MB231 cells. [file mmc3.pdf]

**A**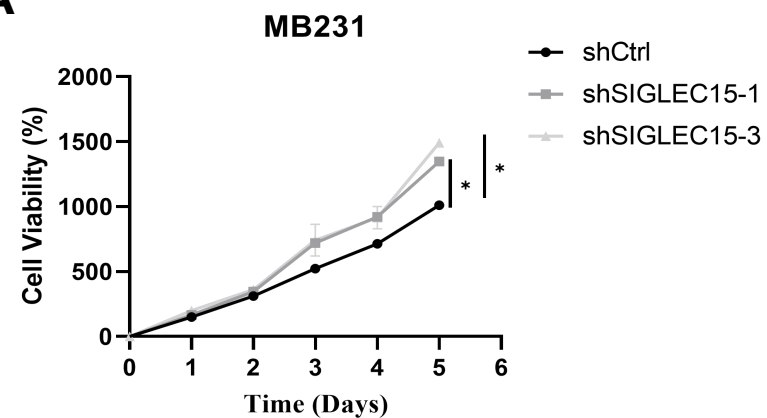**B**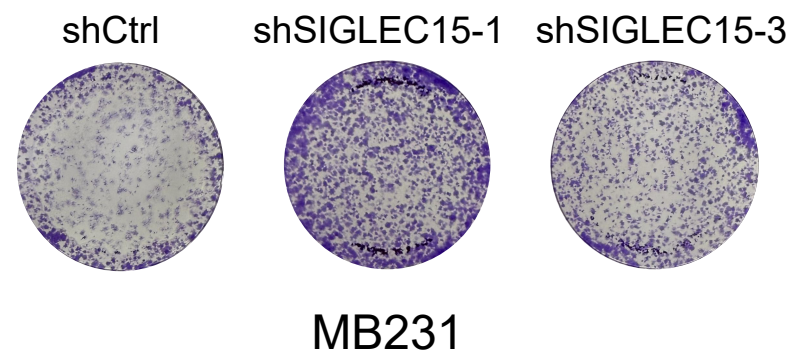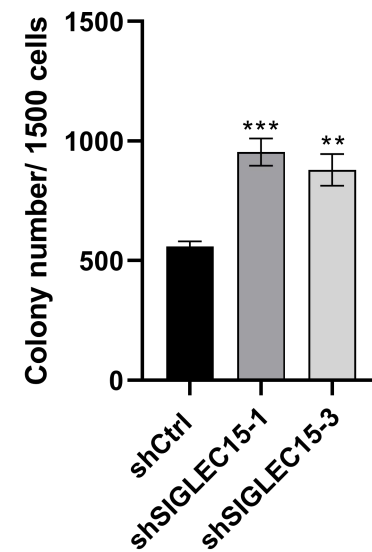**C**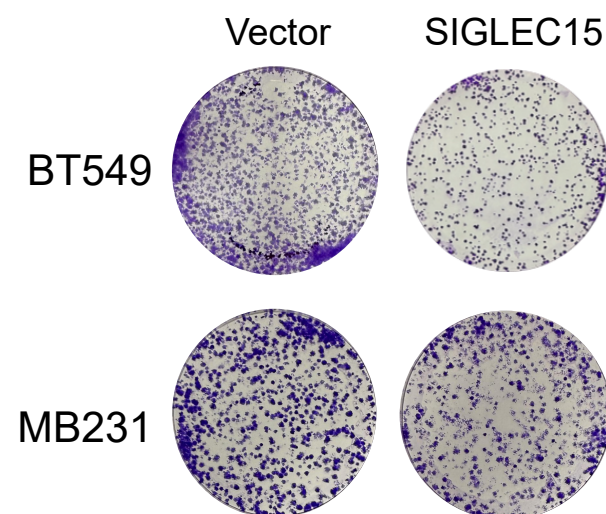**D**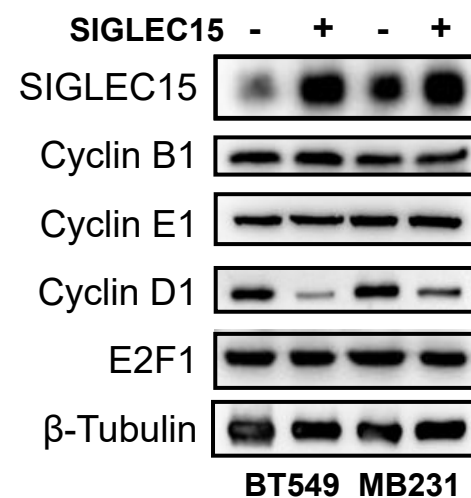**E**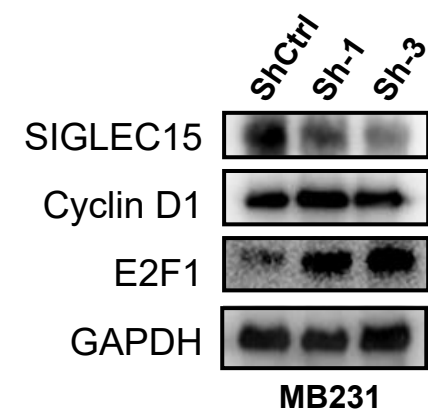**F**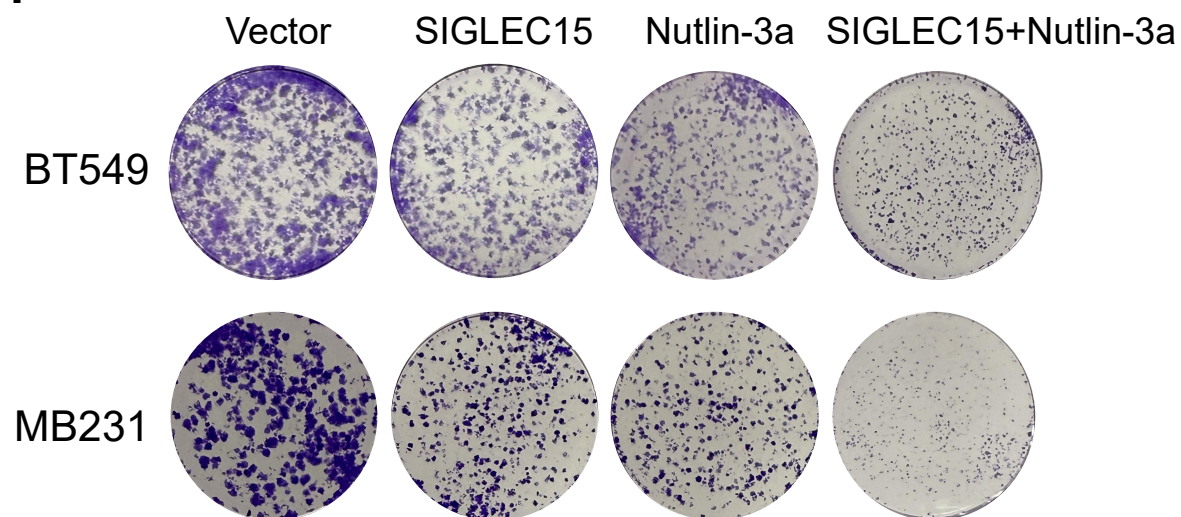**G**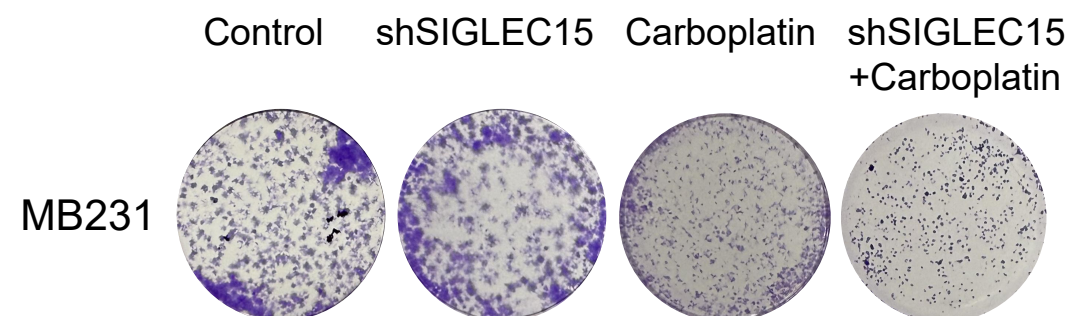

Supplement: Multimedia component 4 — Figure S4 Effects of SIGLEC15 on the proliferation of TNBC cells and drug selection based on SIGLEC15 expression. (A) Comparison of the cell proliferation between the SIGLEC15 knockdown and control group of MB231 cell using the CCK8 method. (B) Comparison of the cell proliferation between the SIGLEC15 knockdown and control group of MB231 cell using the colony formation assay. Quantitative analysis for the colony formation assay results. (C) Comparison of the cell proliferation between the SIGLEC15 overexpression and control group of BT549 and MB231 breast cancer cells using the colony formation assay. (D) Western blot for cell cycle-related proteins in SIGLEC15-overexpression BT549 and MB231 cells. (E) Western blot for cell cycle-related proteins in SIGLEC15-knowdown MB231 cell. (F) Colony formation assay was used to evaluate the therapeutic effect of Nutlin-3a on SIGLEC15-overexpressing BT549 and MB231 cells. (G) Colony formation assays verified the inhibitory effect of carboplatin on SIGLEC15-knockdown MB231 cells. [file mmc4.pdf]
